# Genetic variability, N-glycosylation, and recombination in sublineage 1A of *Betaarterivirus americense* from commercial pig farms in Lima, 2019

**DOI:** 10.3389/fmicb.2026.1803991

**Published:** 2026-05-18

**Authors:** Rony Yunior Cotaquispe Nalvarte, Miriam Legua Barrios, Edgard De la Cruz Vásquez, Julio Cesar Ecos Espino, Erick Llona García, Ayda Liliana Reyes Ruiz, Merici Ingrid Medina Guerrero, Felicita Karina Camargo Paredes, Hilda Victoria Coila De la Cruz, Elvia Mejía Vargas, Cesar Augusto Mendoza Yáñez, Carmen Lucy Cabrel Palomares, Jennifer Toskano Hurtado

**Affiliations:** 1 Universidad Privada de Ciencias y Humanidades, Lima, Peru; 2 Molecular Biology and Clinical Diagnostics Research Group for Emerging Diseases (R&D), Universidad Privada de Ciencias y Humanidades, Lima, Peru; 3 Molecular Research and Biosciences Laboratory (IMBIOS), Department of Research & Development, Corporación Montana S.A., Lima, Peru; 4 Universidad Privada San Juan Bautista, Ica, Peru; 5 Universidad Privada Autónoma de Ica, Ica, Peru; 6 Universidad Nacional San Luis Gonzaga, Ica, Peru; 7 Universidad Nacional José Faustino Sánchez Carrión, Lima, Peru; 8 Hospital Felix Torrealva Gutiérrez, Ica, Peru

**Keywords:** genetic diversity, N-glycosylation, ORF5, RDP4.101, recombination, sublineage 1A

## Abstract

Porcine reproductive and respiratory syndrome virus (PRRSV) is highly variable, and emerging variants have been responsible for severe outbreaks with unprecedented economic losses. Lima represents a major center of pig production; however, the absence of a structured epidemiological surveillance program has facilitated the spread of divergent variants in commercial farms. The present study provides an initial assessment of GP5 variability in a high-risk production setting. The aim of this study was to characterize the genetic variability, N-glycosylation sites, and recombination events within this region. Bioinformatic tools and servers were used for phylogenetic analyses, revealing that all 24 analyzed strains clustered within sublineage 1A, with evidence of divergent variants. Several amino acid substitutions were identified in domains associated with neutralizing and non-neutralizing antibody responses, including A27V/S (12/24), A31T (1/24), N32S/G/R/E (16/24), S34N/T (3/24), S35N/I (6/24), H38L (1/24), L39F (7/24), Q40L/R (6/24), L41Y/V (3/24), I42V (1/24), Y43H (4/24), N44I (4/24), L45R (4/24), T46A (4/24), K58E/V/R (15/24), and S59H/R/N (8/24) in GP5. Nine N-glycosylation patterns (A–I) were identified, with nine putative sites at N30, N32, N33, N34, N35, N44, N50, N51, and N57. Patterns A, B, E, and G exhibited five to six potential glycosylation sites in 12/24 strains. A recombination event was detected in strain RC05088942.1_montana/PERU/2019-R, with RC05088941.1_montana/PERU/2019 as the putative major parent (96.1% similarity) and an unidentified minor parent; however, strain RC05088939.1_montana/PERU/2019 showed the closest phylogenetic relationship. Genetic diversity analysis revealed 172 polymorphic sites. Tajima’s D test yielded a value of −0.84020, which was not statistically significant (*P* > 0.10), indicating that the null hypothesis of neutral evolution could not be rejected. Overall, this study provides the first detailed characterization of ORF5 gene diversity in PRRSV strains detected in pigs suspected of infection from commercial farms in Lima in 2019. Broader sampling with temporal resolution is required to better understand nationwide evolutionary patterns.

## 1 Introduction

Porcine reproductive and respiratory syndrome (PRRS) remains one of the most economically significant diseases affecting the global swine industry due to its substantial impact on animal health and productivity (Holtkamp et al., 2013). The etiological agent, currently classified by the International Committee on Taxonomy of Viruses (ICTV; MSL39.v3, 2023) as *Betaarterivirus americense* (PRRSV-2) and *Betaarterivirus europensis* (PRRSV-1), has been extensively characterized at the molecular and structural levels. These species were named according to the geographic origin of their prototype strains, VR-2332 and Lelystad, respectively (Brinton et al., 2021; Jeong et al., 2025; Zheng et al., 2024).

The viral genome consists of a positive-sense single-stranded RNA molecule containing at least eleven open reading frames (ORFs): ORF1a, ORF1b, ORF2a, ORF2b, ORF3, ORF4, ORF5, ORF5a, ORF6, ORF7, and ORF2TF, in addition to a 3′ untranslated region (3′ UTR) (Brinton et al., 2021; Zheng et al., 2024). ORF1a and ORF1b occupy approximately three-quarters of the genome and encode the replicase polyproteins pp1a and pp1ab. The ORFs located at the 3′ terminal region encode eight structural proteins, including four membrane-associated glycoproteins (GP2a, GP3, GP4, and GP5), three non-glycosylated membrane proteins (E, ORF5a, and M), and the nucleocapsid protein N (Jeong et al., 2025; Zheng et al., 2024).

Among these, ORF5 encodes the GP5 glycoprotein, the major envelope protein and a key determinant of viral infectivity. GP5 is a glycosylated transmembrane protein exposed on the virion surface, involved in receptor binding and recognized as one of the primary targets of the humoral immune response (Jakab et al., 2022; Zhou et al., 2018). It contains critical antigenic domains and multiple N-glycosylation sites associated with viral neutralization. N-glycosylation contributes to immune evasion by modulating epitope accessibility to neutralizing antibodies (Jakab et al., 2022). High genetic variability, driven by point mutations and recombination, promotes the emergence of novel *B. americense* variants and contributes to the increasing diversity of circulating genotypes (Guo et al., 2018; Jakab et al., 2022). Phylogenetic analysis of viral gene sequences has classified PRRSV-1 into four subtypes, whereas PRRSV-2 comprises 11 monophyletic lineages (L1–L11) and multiple sublineages, including 1A–1F and 1H–1J within L1, 5A–5B within L5, 8A–8E within L8, and 9A–9E within L9 (Jantafong et al., 2025; Jeong et al., 2025; Yim-im et al., 2023; Zeller et al., 2024).

In the present study, bioinformatic approaches were used to assess genetic diversity and detect recombination events within the ORF5 gene. Recombination analysis was performed using RDP4 (v4.101), which integrates multiple algorithms (RDP, GENECONV, BootScan, MaxChi, Chimera, SiScan, and 3Seq). Only recombination events supported by multiple methods were retained to ensure statistical robustness (Pellegrini Ferreira et al., 2025). DnaSP v6.12.03 was used to estimate genetic diversity parameters and polymorphic sites (Rozas et al., 2017), while MEGA v6.06 was applied for comparative phylogenetic analyses (Guo et al., 2018).

In Lima, Peru, the molecular detection of sublineage 1A (NADC34-like variant; IA/2014/NADC34) reported by Cotaquispe et al. (2022) 2025 has raised concerns regarding the extent of genetic diversification and the potential contribution of recombination among circulating field strains. However, detailed molecular characterization in this epidemiological context remains limited. Therefore, the aim of this study was to characterize genetic diversity and evaluate recombination events shaping the molecular architecture of field strains. These findings provide novel insights into viral diversity and molecular epidemiology in pigs suspected of PRRS sampled from commercial farms in Lima in 2019.

## 2 Materials and methods

### 2.1 Biological material

Sampling was performed by farm veterinarians using a convenience-based approach due to logistical and budgetary constraints. Sample selection was based on accessibility and the presence of pigs exhibiting clinical signs consistent with PRRS in 2019. A total of 100 blood samples were collected from 12 commercial farms. Each representative submitted an average of nine tubes; however, some samples were excluded for not meeting processing requirements. Samples were transported in sealed dry ice containers to the IMBIOS laboratory (Corporación Montana, Lima, Peru) and centrifuged using an Orto Alresa Consul 21 centrifuge at 2,500 × *g* for 5 min. Serum was transferred into 1.5 mL tubes and stored at −20 °C.

### 2.2 RNA extraction, quantification, and conventional RT-PCR of the ORF5 gene

Viral RNA was extracted using the InnuPREP Virus DNA/RNA Kit (Analytik Jena) following the manufacturer’s instructions. A volume of 200 μL of serum was used per extraction. Inactivated vaccines were included as controls: type 2 (American strain, Nebraska Prime Pac^®^ PRRS) and type 1 (European strain, VP-046 SUIPRABAC^®^ PRRS). RNA purity was assessed by spectrophotometry, with most samples showing an A260/A280 ratio close to 2.0. Extracted RNA was stored at −80 °C.

Reverse transcription was performed in two steps. The first mixture (14.5 μL) contained 5 μL of viral RNA (40 ng/μL), 1 μL of random or gene-specific reverse primers, 1 μL of dNTPs (10 mM), and 7.5 μL of RNase-free water. The mixture was incubated at 65 °C for 5 min, chilled on ice for 1 min, and briefly centrifuged. The second mixture (5.5 μL) contained 4 μL of 5× RT buffer, 0.5 μL of RNase inhibitor (40 U/μL), and 1 μL of OneScript RTase (200 U/μL). Both mixtures were combined and centrifuged. cDNA synthesis was performed at 42 °C for 50 min, preceded by primer annealing at 25 °C for 10 min (random primers only), followed by enzyme inactivation at 85 °C for 5 min. cDNA was stored at −80 °C.

PCR amplification was carried out in a final volume of 20 μL containing 3 μL of cDNA (40 ng/μL), 10 μL of 2× PCR HotStart Master Mix (ABM^®^), 5 μL of nuclease-free water, and 1 μL of each primer (10 pmol/μL): SKA-F (5′-GGTGGGCAACKGTTTTAGCCTGTC-3′) and SKA-R (5′-GGTAAT AGARAAYGCCAAAAGCACC-3′). Primers targeting the ORF5 gene were previously validated for sensitivity, specificity, detection limit, repeatability, and robustness. PCR conditions included an initial denaturation at 94 °C for 5 min, followed by 45 cycles of 94 °C for 45 s, 60 °C for 60 s, and 72 °C for 45 s, with a final extension at 72 °C for 10 min. The expected amplicon size was 723 bp, spanning genomic positions 13.4–14.0 kb (Shin et al., 2021).

### 2.3 Electrophoresis, purification, and sequencing

Positive amplicons (*n* = 24) were visualized by horizontal gel electrophoresis using a multiSUB Choice system (Cleaver Scientific) with 1% molecular-grade agarose. DNA was stained with Safe-Green dye (ABM^®^), and fragment size was estimated using a 100 bp DNA ladder (50 bp–1.5 kb, ABM^®^).

Gels were documented using a UVP UV Solo Touch imaging system (Analytik Jena). Bands of the expected size were excised and purified using the innuPREP nucleic acid purification kit (Analytik Jena). Purified PCR products were sequenced bidirectionally by Macrogen (Seoul, South Korea) using the BigDye^®^ Terminator v3.1 Cycle Sequencing Kit (Life Technologies) on an ABI 3137 XL Genetic Analyzer (Life Technologies).

### 2.4 Phylogenetic analysis of ORF5

Raw chromatograms were inspected and edited using Chromas Lite^®^ v2.6.6. Lineage assignment was based exclusively on the ORF5 gene (603 nt) and analyzed using Nextclade v3.21.0 (Aksamentov et al., 2021) to refine phylogenetic relationships. The dataset included 49 sequences: 24 study strains, nine Peruvian isolates, and 16 reference sequences retrieved from GenBank. Phylogenetic trees were constructed using IQ-TREE v2.4.0 and visualized with FigTree v1.4.4. Maximum likelihood analysis was performed under the GTR nucleotide substitution model. Branch support was assessed using UltraFast bootstrap (1,000 replicates) and SH-aLRT test (1,000 replicates).

### 2.5 Antigenic structure and N-glycosylation analysis of GP5

Amino acid sequences were analyzed using MEGA v6.06 to (Tamura et al., 2013) characterize structural and immunologically relevant domains, including the signal peptide, decoy epitopes I (RHV1) and II, the primary neutralizing epitope, transmembrane regions (I–III), T-cell epitopes (I–II), and the third B-cell epitope. This analysis enabled the identification of amino acid substitutions within key functional domains potentially associated with viral infectivity, pathogenicity, and persistence. Protein length was determined for each strain, and potential N-glycosylation sites (N-X-S/T motifs) were predicted using NetNGlyc 1.0 (Gupta et al., 2002). Signal peptides were predicted using SignalP v4.0 (Petersen et al., 2011).

### 2.6 Genetic diversity analysis: Tajima’s D and DnaSP v6.12.03

Genetic diversity was evaluated under the framework of neutral theory. Tajima’s D (Tajima, 1989) statistic was calculated to compare two estimates of nucleotide diversity (θ) within the dataset. Under neutrality, Tajima’s D is expected to be zero. Deviations from this value may indicate selection or demographic effects (Tajima, 1993). Negative values suggest an excess of low-frequency polymorphisms, consistent with purifying selection or population expansion, whereas positive values indicate balancing selection or population bottlenecks. Tajima’s D and other diversity parameters were calculated using DnaSP v6.12.03.

### 2.7 Recombination detection analysis of ORF5 using RDP4

Aligned sequences were analyzed for recombination signals using RDP4 (v4.101), which implements seven independent methods: RDP (Martin and Rybicki, 2000), GENECONV (Padidam et al., 1999), BootScan (Martin et al., 2005), MaxChi (Smith, 1992), Chimera (Posada, 2002), SiScan (Gibbs et al., 2000), and 3Seq (Lam et al., 2018). Default parameters were applied. Recombination events were considered reliable only when supported by at least four independent methods with associated *p*-values < 0.05, ensuring statistical robustness and minimizing false-positive signals.

## 3 Results

### 3.1 Amplification and sequencing of ORF5

A total of 24 serum samples were analyzed using an in-house RT-PCR assay targeting the ORF5 gene. Primers were validated and standardized using positive controls from both PRRSV species. Amplification yielded a 723 bp fragment (Supplementary Materials 1–4). Sequencing confirmed the presence of the complete coding region, comprising 603 nucleotides and encoding a functional protein of 201 amino acids.

### 3.2 Phylogenetic analysis of the ORF5 gene

**FIGURE 1:**
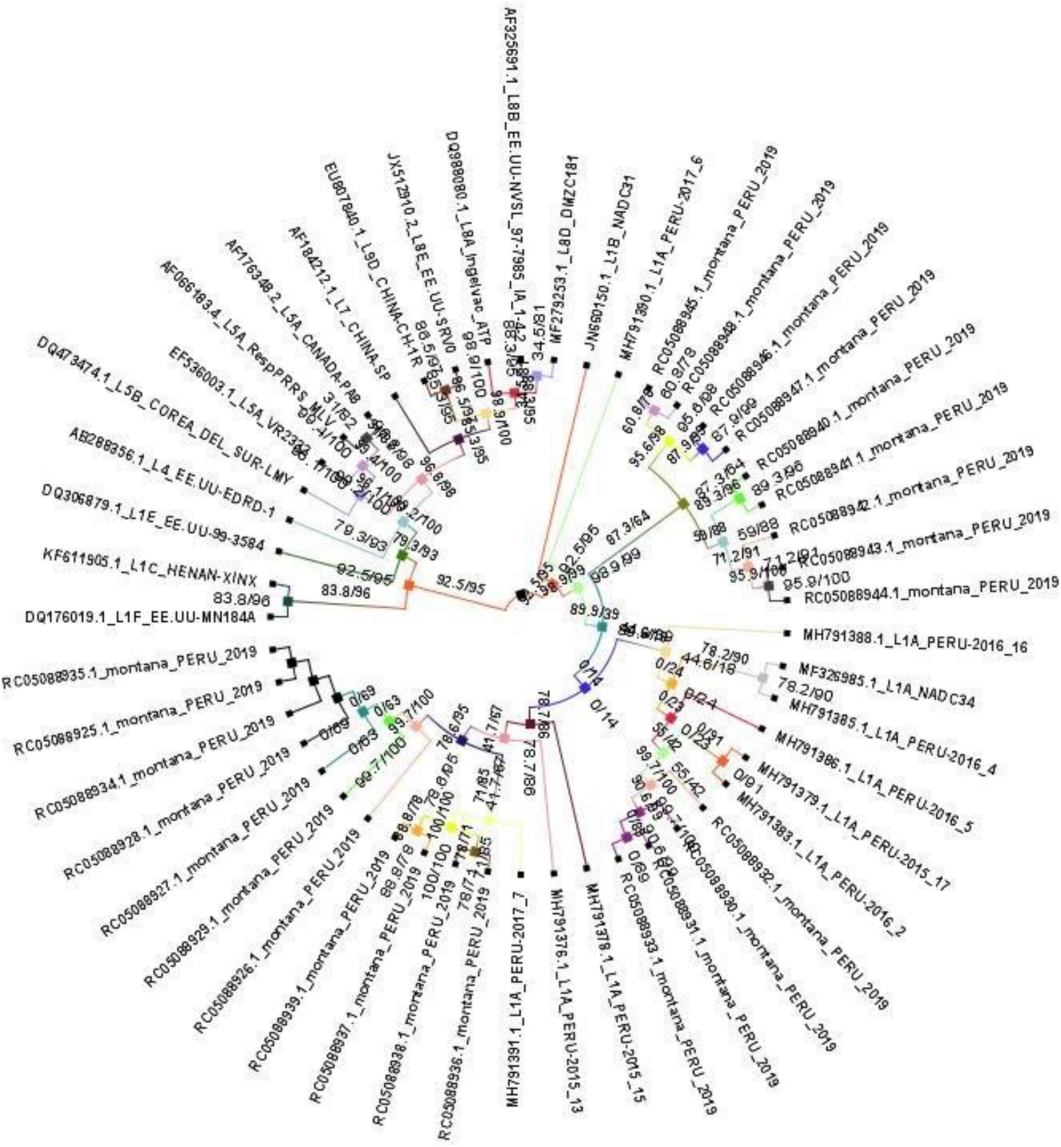
Maximum-likelihood phylogenetic tree of *Betaarterivirus americense* based on the ORF5 gene, generated using IQ-TREE v2.4.0. Lineage clustering of *Betaarterivirus americense*: sublineage 1A (MF326985.1_L1A/NADC34 and 24 study strains), sublineage 1B (JN660150.1_L1B/NADC31), sublineage 1C (KF611905.1_L1C/HENAN-XINX), sublineage 1E (DQ306879.1_L1E/US-99-3584), sublineage 1F (DQ176019.1_L1F/US-MN184A); lineage 4 (AB288356.1_L4/US-EDRD-1); lineage 5: sublineage 5A (AF066183.4_L5A/RespPRRS_MLV and EF536003.1_L5A/VR2332); lineage 7 (AF184212.1_L7/CHINASP); lineage 8: sublineage 8A (DQ988080.1_L8A/Ingelvac_ATP); lineage 9: sublineage 9D (EU807840.1_L9D/CHINA-CH-1R). The phylogenetic analysis was performed using the maximumlikelihood (ML) method under the GTR nucleotide substitution model based on 49 sequences (24 study strains, 9 Peruvian reference strains, and 16 NCBI reference sequences). Branch support was assessed using Ultrafast bootstrap with 1,000 replicates and SH-aLRT with 1,000 replicates.

Phylogenetic relationships were inferred from nucleotide sequence alignments combined with representative reference strains from GenBank (lineages 1, 5, and 8), using Nextclade v3.21.0 (Supplementary Material 5) and IQ-TREE v2.4.0. The resulting topology is shown in Figure 1.

Nine previously reported Peruvian isolates belonging to sublineage 1A (1-7-4/NADC34-like variant) were included for comparative analysis. The phylogeny resolved multiple well-supported branches corresponding to PRRSV-2 lineages, including lineage 1 (sublineages 1A, 1B, 1C, 1E, and 1F), lineage 4, lineage 5 (sublineage 5A), lineage 7, lineage 8 (sublineage 8A), and lineage 9 (sublineage 9D).

All study strains clustered within sublineage 1A, forming a well-defined clade together with Peruvian isolates reported between 2015 and 2017 (Figure 1).

### 3.3 Amino acid diversity of GP5

**FIGURE 2:**
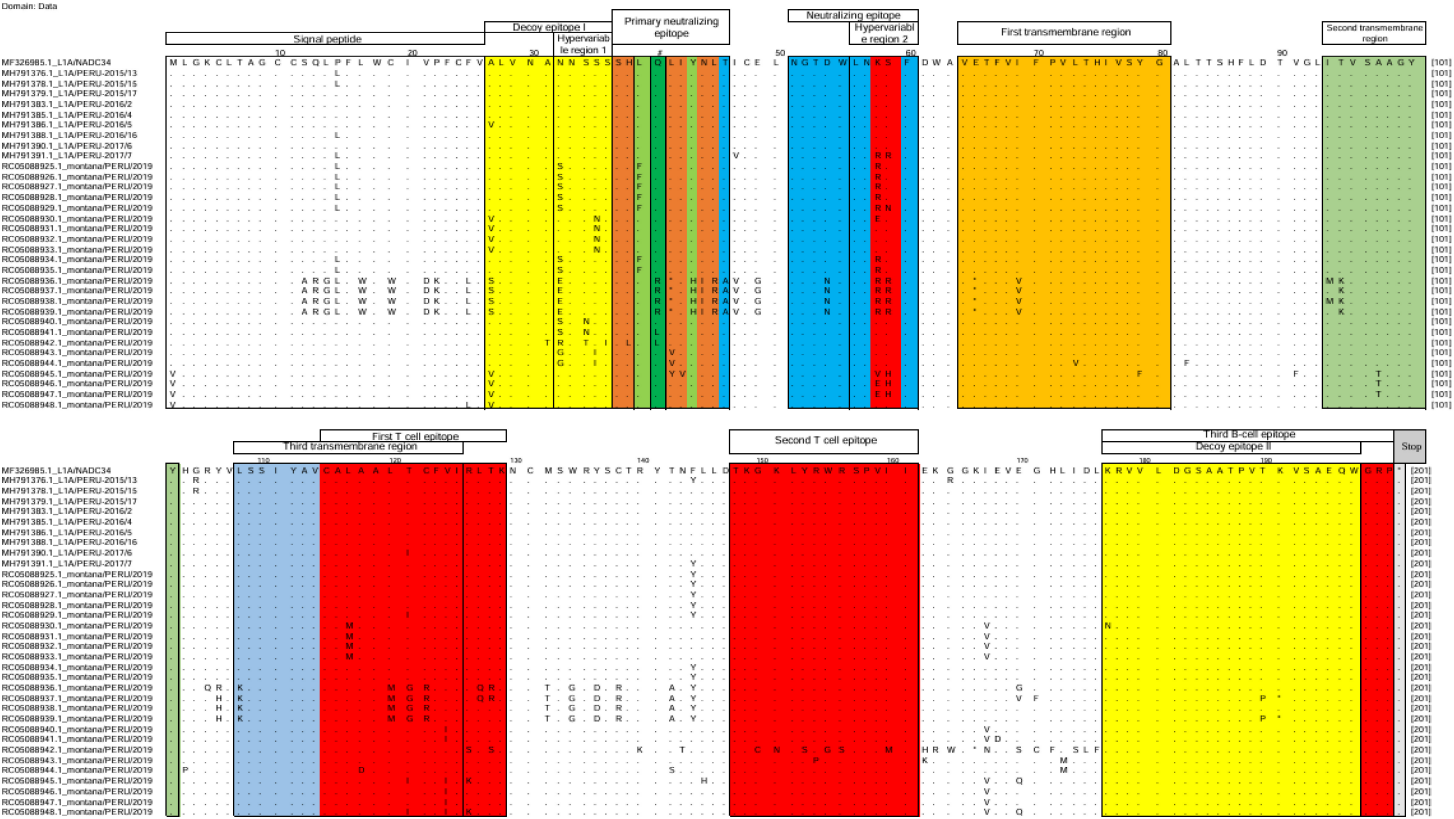
Amino acid alignment of GP5 antigenic regions from 24 *Betaarterivirus americense* sequences within sublineage 1A. Boxed and color-coded regions indicate the structural domains of glycoprotein 5, including antibody neutralizing epitopes. The alignment included 24 field strains, the reference lineage strain MF326985.1_IA/2014/NADC34, and nine representative Peruvian variants of sublineage 1A. Dots indicate conserved amino acid positions, whereas letters represent amino acid substitutions.

Translated sequences (201 amino acids) exhibited the expected structural organization: signal peptide (aa 1–26), decoy epitope I (aa 27–36), decoy epitope II (aa 177–197), primary neutralizing epitope (aa 37–46), adjacent neutralizing epitope (aa 51–60), transmembrane regions I–III (aa 64–80, 94–102, and 108–125), T-cell epitopes (aa 115–129 and 148–161), B-cell epitope (aa 177–200), and a stop codon at position 201 (Figure 2).

Multiple amino acid substitutions were identified within antigenically relevant domains. In the signal peptide region (aa 1–26), substitutions were mostly conservative. Within decoy epitope I (RHV1), substitutions included A27V/S (12/24), A31T (1/24), N32S/G/R/E (16/24), S34N/T (3/24), S35N/I (6/24), and S36I (1/24). In decoy epitope II, substitutions included K177N (1/24) and T190P (2/24), potentially affecting cross-neutralization dynamics. Within the primary neutralizing epitope, key substitutions were observed at positions H38L (1/24), L39F (7/24), Q40L/R (6/24), L41Y/V (3/24), I42V (1/24), Y43H (4/24), N44I (4/24), L45R (4/24), and T46A (4/24). In the adjacent neutralizing epitope (RHV2), substitutions were detected in K58E/V/R (15/24) and S59H/R/N (8/24).

### 3.4 N-glycosylation pattern analysis of GP5

Predictive analysis identified nine distinct N-glycosylation patterns (A–I). A total of nine potential N-glycosylation sites (N-X-S/T motifs) were detected: N30, N32, N33, N34, N35, N44, N50, N51, and N57.

Key glycosylation sites associated with viral neutralization (N33, N44, and N51) were present in most strains. Notably, 20 out of 24 isolates contained both N44 and N51. All isolates exhibited at least three predicted N-glycosylation sites. Strains 40 and 41 showed the highest number of predicted glycosylation sites (Table 1).

**TABLE 1:** Locations and patterns of putative N-glycosylation sites in GP5 among 24 *Betaarterivirus americense* sequences from Lima, Peru.

Sequence data	GP5 (aa)	Signal peptide	Glycosylation pattern	N-glycosylation sites									Strains with N-glycosylation sites
				N30	N32	N33	N34	N35	N44	N50	N51	N57	
Sequences 25–48, sublineage L1A	201	1–31	A	**+**		**+**			**+**		+	+	RC05088925.1, RC05088926.1 RC05088927.1, RC05088928.1 RC05088934.1, RC05088935.1
			B		**+**			**+**	**+**		+	+	RC05088930.1, RC05088931.1 RC05088932.1, RC05088933.1
			C			**+**				**+**			RC05088936.1, RC05088937.1 RC05088938.1, RC05088939.1
			D		**+**	**+**			**+**		+		RC05088945.1; RC05088946.1; RC05088947.1
			E	**+**		**+**	**+**		**+**		+	+	RC05088940.1; RC05088941.1
			F						**+**		+	+	RC05088943.1; RC05088944.1
			G		**+**	**+**			**+**		+	+	RC05088948.1
			H			**+**			**+**		+	+	RC05088942.1
			I	**+**		**+**			**+**		+		RC05088929.1

aa, amino acids.

Signal peptide cleavage prediction using SignalP v4.0 indicated a cleavage site between residues 31 and 32, consistent with canonical viral protein processing (Table 1).

### 3.5 Genetic diversity and Tajima’s D analysis

Genetic diversity analysis was performed on 24 aligned sequences spanning 603 bp, with no missing data or gaps. A total of 172 polymorphic sites and 200 mutations were identified. Nucleotide diversity was estimated at π = 0.07023, with an average number of nucleotide differences of *k* = 42.35.

Tajima’s D test was applied to assess deviations from the neutral evolution model. The estimated value was negative (*D* = −0.84020); however, it was not statistically significant (*P* > 0.10), indicating that the null hypothesis of neutral evolution cannot be rejected (Table 2).

**TABLE 2:** Genetic diversity of 24 *Betaarterivirus americense* isolates based on DnaSP v6.12.03 analysis of pig blood samples from Lima, Peru.

Strains	Sequence	ORF 5 (GP5)	C.S.	Variable sites	P. I. S	U. V. S	Tajima’s D	Nucleotide diversity	Average nucleotide differences (k)
24	Nt (aa)	603 (201)	431 (118)	172 (82)	119 (51)	53 (31)	*D* = −0.84020	π = 0.07023	*k* = 42.35

Nt, nucleotide; aa, amino acid; C.S., conserved sites; P.I.S., parsimony-informative sites; U.V.S, unique variable sites.

### 3.6 Recombination analysis of ORF5 using RDP4

Recombination analysis identified a statistically significant recombination event in strain RC05088942.1_montana/PERU/2019-R. The event was consistently detected by all seven algorithms implemented in RDP4 (RDP, GENECONV, BootScan, MaxChi, Chimera, SiScan, and 3Seq), meeting conservative criteria for recombination inference.

Topological incongruence between phylogenies reconstructed from non-recombinant regions (1–439 and 550–603 nt) and the recombinant region (440–549 nt) supported this finding (Supplementary Materials 2, 3–6). The major parental sequence corresponded to strain RC05088941.1_montana/PERU/2019 in the non-recombinant regions. The minor parent was not directly identified in the dataset; however, strain RC05088939.1_montana/PERU/2019 showed the closest phylogenetic affinity within the recombinant region.

Statistical support for recombination was significant across all methods: RDP (3.637 × 10^–4^), GENECONV (9.535 × 10^–6^), BootScan (8.859 × 10^–6^), MaxChi (7.365 × 10^–6^), Chimera (2.474 × 10^–6^), SiScan (6.495 × 10^–11^), and 3Seq (9.731 × 10^–9^) (Table 3). The concordance among seven independent methods strongly supports the robustness of the detected recombination event.

**TABLE 3:** Genetic recombination event detected in the RC05088942.1_montana/PERU/2019-R strain using RDP4.101.

Recombination events	Methods	Breakpoints (nt)		Major parent (similarity)	Minor parent (similarity)	*P*-value
		Start	End			
Strain RC05088942.1-R	RDP	440	549	RC05088941.1_montana/PERU/2019; (nt 1–439 and 550–603) (96.1% similarity)	Unknown Best candidate RC05088939.1_montana/PERU/2019 (nt 440–549)	3.637 × 10^–04^
	GENECONV					9.535 × 10^–06^
	BootScan					8.859 × 10^–06^
	MaxChi					7.365 × 10^–06^
	Chimera					2.474 × 10^–06^
	SIScan					6.495 × 10^–11^
	3Seq					9.731 × 10^–09^

## 4 Discussion

All 24 sequences analyzed in this study were classified as *Betaarterivirus americense*. Phylogenetic inference was performed using FASTA sequences with accession numbers aligned against globally relevant epidemic strains retrieved from Nextclade v3.21.0 (Supplementary Material 5). In addition, maximum likelihood phylogenetic analysis enabled the identification of lineages 1, 5, and 8. The GP5 phylogeny confirmed that all study strains clustered within lineage 1, sublineage 1A (NADC34-like variant, MF326985.1_L1A), with notable variability observed among strains 36, 37, 38, 39, and 42 (Figure 1; Supplementary Material 6).

Structural analysis of GP5 indicates that antigenic domains play critical roles in immune recognition. Both synonymous and non-synonymous substitutions were identified in key immunologically relevant regions, including decoy epitopes I (hypervariable region 1) and II, as well as the primary and adjacent neutralizing epitopes (hypervariable region 2) (Guo et al., 2018; Jones et al., 1992). Compared with the reference strain MF326985.1_IA/2014/NADC34, varying degrees of substitutions were observed in the signal peptide and antigenic domains.

Within decoy epitope I (RHV1), substitutions were particularly frequent, including A27V/S (12/24), A31T (1/24), N32S/G/R/E (16/24), S34N/T (3/24), S35N/I (6/24), and S36I (1/24). This pattern is consistent with the rapid induction of non-neutralizing antibody responses (Hong et al., 2019; Wang et al., 2025). These decoy epitopes (aa 27–36 and aa 177–197) are thought to delay the generation of effective neutralizing antibodies (Zhou et al., 2018).

Within the primary neutralizing epitope, substitutions at positions H38L, L39F, Q40L/R, L41Y/V, I42V, Y43H, N44I, L45R, and T46A indicate substantial variability. Previous studies have identified residues 38, 42, 43, and 44 as critical determinants of neutralization, while positions 39–41 are involved in antibody binding, with residue 39 frequently used for variant classification (Faaberg et al., 2006; Zhou et al., 2018). Mutations in these regions have been associated with reduced sensitivity of PRRSV-2 to neutralizing antibodies *in vivo* (Chen et al., 2017; Nguyen et al., 2022). Although neutralization assays were not performed in this study, targeted functional analyses are warranted to evaluate the impact of these substitutions on viral pathogenicity and vaccine escape.

In the adjacent neutralizing epitope (RHV2), substitutions K58E/V/R (15/24) and S59H/R/N (8/24) were also observed and may contribute to immune evasion, as previously suggested (Nguyen et al., 2022). It is important to note that the amino acid substitution analysis presented here is descriptive. Functional validation through *in vitro* and *in vivo* neutralization assays is required to quantify the impact of these mutations on antibody sensitivity, particularly in comparison with strains used in commercial vaccines.

N-glycosylation analysis of GP5, predicted using NetNGlyc 1.0, revealed nine potential glycosylation sites (N30, N32, N33, N34, N35, N44, N50, N51, and N57) across the 24 strains, forming nine distinct glycosylation patterns (A–I). Glycosylation at key sites associated with neutralization (N33, N44, and N51) was frequently observed. Strains 40 and 41 exhibited the highest number of predicted glycosylation sites, consistent with previous reports (Jakab et al., 2022; Zhang et al., 2017). Increased N-glycosylation has been proposed to shield neutralizing epitopes and reduce immunogenicity (Ansari et al., 2006). However, functional studies are required to determine its impact on epitope exposure, immune recognition, and viral pathogenicity.

Recombination analysis identified a statistically significant event in strain RC05088942.1_montana/PERU/2019-R within nucleotide positions 440–549, corresponding to the mid-C-terminal region of ORF5, which encompasses functionally relevant GP5 domains. The major and minor parental sequences were inferred as RC05088941.1_montana/PERU/2019 and RC05088939.1_montana/PERU/2019, respectively. Although overall nucleotide similarity with the major parent was approximately 96.1%, the recombinant segment showed higher phylogenetic affinity with the minor parent, supporting genomic fragment exchange (Martin et al., 2015; Pellegrini Ferreira et al., 2025; Wang et al., 2019).

Maximum likelihood trees constructed for non-recombinant regions (1–439 and 550–603 nt) and the recombinant region (440–549 nt) revealed clear topological incongruence. In the non-recombinant regions, strain RC05088942.1_montana/PERU/2019-R clustered with the major parent, whereas in the recombinant region it clustered with RC05088939.1_montana/PERU/2019. This pattern is consistent with a mosaic genome structure and intralineage homologous recombination. Although homoplasy and convergent evolution cannot be fully excluded, the consistent detection across seven independent algorithms, well-defined breakpoints, and strong topological evidence support a genuine recombination event.

It is important to acknowledge that recombination inference based on a relatively short ORF5 fragment (603 nt) has inherent limitations. Ideally, these findings should be validated using full-length genomes or longer genomic regions to improve resolution and accuracy.

Genetic diversity analysis revealed 172 polymorphic sites and 200 mutations. Nucleotide diversity (π = 0.07023) and the average number of nucleotide differences (*k* = 42.35) support a high level of genetic variability. Tajima’s D test yielded a negative value (*D* = −0.84020), suggesting an excess of low-frequency variants. This pattern is theoretically consistent with recent population expansion or purifying selection. However, the result was not statistically significant (*P* > 0.10), indicating that the null hypothesis of neutral evolution cannot be rejected. Therefore, although the negative trend may reflect underlying demographic or selective processes, the evidence is insufficient to support definitive conclusions.

Overall, these findings demonstrate substantial genetic diversity in the GP5 gene among PRRSV strains detected in pigs suspected of infection from commercial farms in Lima in 2019. This highlights the need for broader sampling with temporal resolution to better understand viral evolution at the national level. The present study does not allow estimation of prevalence, lineage distribution, spatiotemporal transmission patterns, or associated risk factors, which should be addressed in future investigations.

## 5 Conclusion

1. Robust phylogenetic trees were inferred using maximum likelihood analysis of 24 strains, all of which clustered within sublineage 1A. Notable divergence was observed in strains 36, 37, 38, 39, and 42 from pigs suspected of PRRS sampled in commercial farms in Lima in 2019.
2. Significant amino acid substitutions were identified in key antigenic regions of GP5. These changes are potentially associated with both neutralizing and non-neutralizing antibody responses, including N32S/G/R/E (16/24), S34N/T (3/24), S35N/I (6/24), H38L (1/24), L39F (7/24), Q40L/R (6/24), L41Y/V (3/24), I42V (1/24), Y43H (4/24), N44I (4/24), L45R (4/24), T46A (4/24), K58E/V/R (15/24), and S59H/R/N (8/24).
3. Nine distinct N-glycosylation patterns (A–I) were identified across the 24 strains, with nine putative glycosylation sites (N30, N32, N33, N34, N35, N44, N50, N51, and N57). Patterns A, B, E, and G exhibited five to six glycosylation sites in 12 of the 24 isolates, particularly in strains RC05088940.1 and RC05088941.1.
4. A statistically significant recombination event was detected in strain RC05088942.1_montana/PERU/2019-R. The inferred major parent was RC05088941.1_montana/PERU/2019 (96.1% similarity), whereas the minor parent was not directly identified but showed the closest phylogenetic affinity with RC05088939.1_montana/PERU/2019.
5. Genetic diversity analysis of 24 ORF5 sequences (603 nt) identified 172 polymorphic sites. Nucleotide diversity (π = 0.07023) and the average number of nucleotide differences (*k* = 42.35) indicated high variability. Tajima’s D value was negative (*D* = −0.84020) but not statistically significant, suggesting population dispersion without rejecting the null hypothesis of neutral evolution in PRRS-suspected pigs sampled in commercial farms in Lima in 2019.

## Data Availability

The original contributions presented in this study are included in this article/Supplementary material, further inquiries can be directed to the corresponding author.

## Supplementary material

Supplementary Material 1 ORF5 sequences from 24 study strains, 9 Peruvian reference strains, and 46 sequences retrieved from GenBank.

Supplementary Material 2 ORF5 gene sequences from sublineage 1A used for recombination analysis with RDP4 (v4.101).

Supplementary Material 3 Sequence data submitted to GenBank under BankIt identifiers 25–48.

Supplementary Material 4 In-house conventional RT-PCR assay targeting the ORF5 gene.

Supplementary Material 5 Phylogenetic tree of *Betaarterivirus americense* based on GP5 sequences, generated using Nextclade v3.21.0.

Supplementary Material 6 Genomic recombination analysis of strain RC05088942.1_montana/PERU/2019-R of porcine reproductive and respiratory syndrome virus (PRRSV) from commercial farms in Lima, 2019.
