# Supplementary material for: Genetic variability, N-glycosylation, and recombination in sublineage 1A of Betaarterivirus americense from commercial pig farms in Lima, 2019
Source: Front Microbiol. 2026 May 18;17:1803991. doi: 10.3389/fmicb.2026.1803991 (PMC13224472; doi:10.3389/fmicb.2026.1803991)
Supplement: Supplementary Material 1 — ORF5 sequences from 24 study strains, 9 Peruvian reference strains, and 46 sequences retrieved from GenBank. [file Supplementary_file_1.pdf]

>RC05088948.1_montana/PERU/2019

GTGTTGGGGAAATGCTTGACCGCGGGCTGCTGCTCGCAATTGCCTTTTTTGTGGTGTATCGTGCCGTTCTGT
CTTGTTGTGCTCGTCAACGCCAACAACAGCAGCAGCTCCCATTTACAGTTGATTTATAACCTGACGATATGT
GAGCTGAATGGCACAGATTGGCTAAACAAAAGTTTTGATTGGGCGGTGGAGACCTTTGTCATCTTTCCTGT
GTTGACTCATATTGTCTCCTATGGCGCCCTTACCACCAGTCATTTCCTTGACACAGTCGGCCTGATCACCGTG
TCTGCCGCCGGATATTACCACGGGCGGTATGTCTTGAGTAGCATTTACGCCGTCTGCGCCTTAGCCGCGTTA
ATTTGCTTCATCATCAAGCTAACAAAAAATTGTATGTCCTGGCGTTACTCATGCACCAGGTACACTAATTTTC
TTCTGGACACCAAGGGCAAACTCTATCGTTGGCGGTCTCCTGTCATCATAGAGAAAGGGGGTAAAGTTGAG
GTCCAAGGTCACCTGATAGACCTCAAAAGAGTTGTGCTTGACGGTTCCGCGGCTACCCCTGTAACCAAAGTT
TCAGCGGAACAATGGGGTCGTCCTTAG

>RC05088947.1_montana/PERU/2019

GTGTTGGGGAAATGCTTGACCGCGGGCTGCTGCTCGCAATTGCCTTTTTTGTGGTGTATCGTGCCGTTCTGT
TTTGTTGTGCTCGTCAACGCCAACAACAGCAGCAGCTCCCATTTGCAGTTGATTTATAACCTGACGATATGT
GAGCTGAATGGCACAGATTGGCTAAACGAACATTTTGATTGGGCGGTGGAGACCTTTGTCATCTTTCCTGTG
TTGACTCACATTGTCTCCTATGGTGCCCTCACCACCAGCCATTTCCTTGACACAGTCGGCCTGATCACCGTGT
CTACCGCCGGATATTACCACGGGCGGTATGTCTTGAGTAGCATTTACGCCGTCTGCGCCCTAGCTGCGTTAA
CTTGCTTCATCATCAGGCTAACGAAAAACTGTATGTCCTGGCGTTACTCATGCACCAGGTACACTAATTTTCT
TCTGGACACCAAGGGCAAACTCTATCGTTGGCGGTCTCCTGTCATCATAGAGAAAGGGGGTAAAGTTGAGG
TCGAAGGTCACCTTATCGACCTCAAGAGAGTTGTACTTGACGGTTCCGCGGCTACCCCTGTAACCAAAGTTT
CAGCGGAACAATGGGGTCGTCCTTAG

>RC05088946.1_montana/PERU/2019

GTGTTGGGGAAATGCTTGACCGCGGGCTGCTGCTCGCAATTGCCTTTTTTGTGGTGTATCGTGCCGTTCTGT
TTTGTTGTGCTCGTCAACGCCAACAACAGCAGCAGCTCCCATTTACAGTTGATTTATAACCTGACGATATGTG
AGCTGAATGGCACAGATTGGCTAAACGAACATTTTGATTGGGCGGTGGAGACCTTTGTCATCTTTCCTGTGT
TGACTCACATTGTCTCCTATGGTGCCCTCACCACCAGCCATTTCCTTGACACAGTCGGCCTGATCACCGTGTC
TACCGCCGGATATTACCACGGGCGGTATGTCTTGAGTAGCATTTACGCCGTCTGCGCCCTAGCTGCGTTAAC
TTGCTTCATCATCAGGCTAACGAAAAACTGTATGTCCTGGCGTTACTCATGCACCAGGTACACTAATTTTCTT
CTGGACACCAAGGGCAAACTCTATCGTTGGCGGTCTCCTGTCATCATAGAGAAAGGGGGTAAAGTTGAGGT
CGAAGGTCACCTGATCGACCTCAAGAGAGTTGTACTTGACGGTTCCGCGGCTACCCCTGTAACCAAAGTTTC
AGCGGAACAATGGGGTCGTCCTTAG

>RC05088945.1_montana/PERU/2019

GTGTTGGGGAAATGCTTGACCGCGGGCTGCTGCTCGCAATTGCCTTTTTTGTGGTGTATCGTGCCGTTCTGT
TTTGTTGTGCTCGTCAACGCCAACAACAGCAGCAGCTCCCATTTACAGTATGTTTATAACCTGACGATATGTG
AGCTGAATGGCACAGATTGGCTAAACGTTCATTTTGATTGGGCGGTGGAGACCTTTGTCATCTTTCCTGTGT
TGACTCACATTGTCTCCTTTGGTGCCCTTACCACCAGCCATTTCCTTGACACATTCGGCCTGATCACCGTGTCT
ACCGCCGGATATTACCACGGGCGGTATGTCTTGAGTAGCATTTACGCCGTCTGCGCCCTAGCCGCGTTAATT
TGCTTCATCATCAAGCTAACAAAAAATTGTATGTCCTGGCGTTACTCATGCACCAGGTACACTAATTTTCATC
TGGACACCAAGGGCAAACTCTATCGTTGGCGGTCTCCTGTCATCATAGAGAAAGGGGGTAAAGTTGAGGTC
CAAGGTCACCTGATAGACCTCAAAAGAGTTGTGCTTGACGGTTCCGCGGCTACCCCTGTAACCAAAGTTTCA
GCGGAACAATGGGGTCGTCCTTAG

>RC05088944.1_montana/PERU/2019

ATGTTGGGGAAATGCTTGACCGCGGGCTGCTGCTCGCAATTGCCTTTTTTGTGGTGTATCGTGCCGTTCTGT
TTTGTTGCGCTCGTCAACGCCGGCAACAGCATCAGCTCCCATTTACAGGTGATTTATAACCTGACGATATGT
GAGCTGAATGGCACAGATTGGCTAAATAAAAGCTTTGATTGGGCGGTGGAGACCTTTGTTATCTTTCCTGTG
GTGACTCATATTGTCTCCTATGGCGCCTTCACCACCAGCCATTTCCTTGACACAGTCGGCCTGATCACCGTGT
CTGCCGCCGGATATTACCCCGGACGGTATGTTTTGAGTAGCATTTACGCCGTTTGCGCCCTGGATGCGTTAA
CTTGCTTCGTCATCAGGCTAACAAAAAATTGTATGTCCTGGCGTTATTCATGCACCAGGTACAGTAATTTTCT
TCTGGACACCAAGGGCAAACTCTATCGTTGGCGGTCTCCTGTCATCATAGAGAAAGGGGGTAAAATAGAG
GTAGAAGGTCACATGATCGACCTCAAGAGAGTTGTACTTGACGGTTCCGCGGCTACCCCTGTAACCAAAGT
TTCAGCGGAACAATGGGGTCGTCCTTAG

>RC05088943.1_montana/PERU/2019

ATGTTGGGGAAATGCTTGACCGCGGGCTGCTGCTCGCAATTGCCTTTTTTGTGGTGTATCGTGCCGTTCTGT
TTTGTTGCGCTCGTCAACGCCGGCAACAGCATCAGCTCCCATTTACAGGTGATTTATAACCTGACGATATGT
GAGCTGAATGGCACAGATTGGCTAAATAAAAGCTTTGATTGGGCGGTGGAGACCTTTGTTATCTTTCCTGTG
TTGACTCATATTGTCTCCTATGGCGCCCTCACCACCAGCCATTTCCTTGACACAGTCGGCCTGATCACCGTGT
CTGCCGCCGGATATTACCACGGACGGTATGTTTTGAGTAGCATTTACGCCGTCTGCGCCCTGGCTGCATTAA
CTTGCTTCGTCATCAGGCTAACAAAAAATTGTATGTCCTGGCGTTATTCATGCACCAGGTACACTAATTTTCT
TCTGGACACCAAGGGCAAACTCTATCCTTGGCGGTCTCCTGTCATCATAAAGAAAGGGGGGAAAATAGAGG
TAGAAGGTCACATGATCGACCTCAAGAGAGTTGTACTTGACGGTTCCGCGGCTACCCCTGTAACCAAAGTTT
CAGCGGAACAATGGGGTCGTCCTTAG

>RC05088942.1_montana/PERU/2019

ATGTTGGGGAAATGCTTGACCGCGGGCTGCTGCTCGCAATTGCCTTTTTTGTGGTGTATCGTGCCATTCTGT
TTTGTTGCGCTCGTCAACACCCGCAACACCAGCATCTCCCTTTTACTTTTGATTTATAACCTGACGATATGTGA
GCTGAATGGCACAGATTGGCTAAATAAAAGCTTTGATTGGGCGGTGGAGACCTTTGTTATCTTTCCTGTGTT
GACTCATATTGTCTCCTATGGCGCCCTCACCACCAGCCATTTCCTTGACACAGTCGGCCTGATCACCGTGTCT
GCCGCCGGATATTACCACGGACGGTATGTTTTAAGTAGCATTTACGCCGTCTGCGCCCTGGCTGCGTTAACT
TGCTTCGTCATCTCGCTATCTAAAAATTGTATGTCCTGGCGTTATTCTTGCACCAAGTACACTACTTTTCTTCT
GGACACCAAGTGCAATCTCTCTCGCGGAAGTTCTCCTGTGATGATACACCGCTGGGGTTAAAACGAAGTCT
CATGTTTCCTGTCCCTCTTCAAAAGAGTTGTGCTTGACGGTTCCGCGGCTACCCCTGTAACCAAAGTTTCAGC
GGAACAATGGGGTCGTCCTTAG

>RC05088941.1_montana/PERU/2019

ATGTTGGGGAAATGCTTGACCGCGGGCTGCTGCTCGCAATTGCCTTTTTTGTGGTGTATCGTGCCGTTCTGT
TTTGTTGCGCTCGTCAACGCCAGCAACAACAGCAGCTCCCATTTACTGTTGATTTATAACCTGACGATATGTG
AGCTGAACGGCACAGATTGGCTAAATAAAAGTTTTGATTGGGCGGTGGAGACCTTTGTTATCTTTCCTGTGT
TGACTCATATTGTCTCCTATGGCGCCCTCACCACCAGCCATTTCCTTGACACAGTCGGCCTGATCACCGTGTC
TGCCGCCGGATATTACCACGGACGGTATGTTTTGAGTAGCATTTACGCCGTCTGCGCCCTGGCTGCGTTAAC
TTGCTTCATCATCAGGCTAACAAAAAATTGTATGTCCTGGCGTTACTCATGCACCAGGTACACTAATTTTCTT
CTGGACACCAAGGGCAAACTCTATCGTTGGCGGTCTCCTGTCATCATAGAGAAAGGGGGTAAAGTTGACGT
CGAAGGTCACCTGATCGACCTCAAGAGAGTTGTACTTGACGGTTCCGCGGCTACCCCTGTAACCAAAGTTTC
AGCGGAACAATGGGGTCGTCCTTAG

>RC05088940.1_montana/PERU/2019

ATGTTGGGGAAATGCTTGACCGCGGGCTGCTGCTCGCAATTGCCTTTTTTGTGGTGTATCGTGCCGTTCTGT
TTTGTTGCGCTCGTCAACGCCAGCAACAACAGCAGCTCCCATTTACAGTTGATTTATAACCTGACGATATGT
GAGCTGAACGGCACAGATTGGCTAAATAAAAGTTTTGATTGGGCGGTGGAGACCTTTGTTATCTTTCCTGTG
TTGACTCATATTGTCTCCTATGGCGCCCTCACCACCAGCCATTTCCTTGACACAGTCGGCCTGATCACCGTGT
CTGCCGCCGGATATTACCACGGACGGTATGTTTTGAGTAGCATTTACGCCGTCTGCGCCCTGGCTGCGTTAA
CTTGCTTCATCATCAGGCTAACAAAAAATTGTATGTCCTGGCGTTACTCATGCACCAGGTACACTAATTTTCT
TCTGGACACCAAGGGCAAACTCTATCGTTGGCGGTCTCCTGTCATCATAGAGAAAGGGGGTAAAGTTGAGG
TCGAAGGTCACCTGATCGACCTCAAGAGAGTTGTACTTGACGGTTCCGCGGCTACCCCTGTAACCAAAGTTT
CAGCGGAACAATGGGGTCGTCCTTAG

>RC05088939.1_montana/PERU/2019

ATGTTGGGGAAATGCTTGACCGCGGGCTGCTGCGCGCGGGGGCTTTTTTGGTGGTGGATCGATAAGTTCTG
TTTGGTTTCGCTCGTCAATGCCGAGAACAGCAGCAGCTCCCATTTAAGATAAATCCATATTCGGGCGGTATG
CGGTCTGAATGGCACAAATTGGCTAAATAGAAGGTTTGATTGGGCGGTGTAGACCTTTGTTGTCTTTCCTGT
GTTGACTCATATTGTCTCCTATGGCGCCCTCACCACCAGCCATTTCCTTGACACAGTCGGTTTGATTAAGGTG
TCTGCCGCCGGATATTACCACGGGCGGCATGTCAAAAGTAGCATTTACGCCGTGTGCGCCCTGGCTGCGAT
GGGCCGATTTGTCATCAGACTAACAAAAAATTGCACCTCCGGGCGGGACTCACGCACCAGATACGCTAACT
ATCTTCTGGACACCAAGGGCAAACTATATCGTTGGCGGTCTCCTGTCATCATAGAGAAAGGGGGTAAAATT
GAGGTCGAAGGTCACCTGATCGACCTCAAGAGAGTTGTGCTTGACGGTTCCGCGGCGACTCCTGTACCCTA
AGTTTCAGCGGAACAATGGGGTCGTCCTTAG

>RC05088938.1_montana/PERU/2019

ATGTTGGGGAAATGCTTGACCGCGGGCTGCTGCGCGCGGGGGCTTTTTTGGTGGTGGATCGATAAGTTCTG
TTTGGTTTCGCTCGTCAATGCCGAGAACAGCAGCAGCTCCCATTTAAGATAAATCCATATTCGGGCGGTATG
CGGTCTGAATGGCACAAATTGGCTAAATAGAAGGTTTGATTGGGCGGTGTAGACCTTTGTTGTCTTTCCTGT
GTTGACTCATATTGTCTCCTATGGCGCCCTCACCACCAGCCATTTCCTTGACACAGTCGGTTTGATGAAGGTG
TCTGCCGCCGGATATTACCACGGGCGGCATGTCAAAAGTAGCATTTACGCCGTGTGCGCCCTGGCTGCGAT
GGGCCGATTTGTCATCAGACTAACAAAAAATTGTACCTCCGGGCGGGACTCACGCACCAGATACGCTAACT
ATCTTCTGGACACTAAGGGCAAACTATATCGTTGGCGGTCTCCTGTCATCATAGAGAAAGGGGGTAAAATT
GAGGTCGAAGGTCACCTGATCGACCTCAAGAGAGTTGTGCTTGACGGTTCCGCGGCGACTCCTGTAACCAA
AGTTTCAGCGGAACAATGGGGTCGTCCTTAG

>RC05088937.1_montana/PERU/2019

ATGTTGGGGAAATGCTTGACCGCGGGCTGCTGCGCGCGGGGGCTTTTTTGGTGGTGGATCGATAAGTTCTG
TTTGGTTTCGCTCGTCAATGCCGAGAACAGCAGCAGCTCCCATTTAAGATAAATCCATATTCGGGCGGTATG
CGGTCTGAATGGCACAAATTGGCTAAATAGAAGGTTTGATTGGGCGGTGTAGACCTTTGTTGTCTTTCCTGT
GTTGACTCATATTGTCTCCTATGGCGCCCTCACCACCAGCCATTTCCTTGACACAGTCGGTTTGATTAAGGTG
TCTGCCGCCGGATATTACCACGGACGGCATGTCAAAAGTAGCATTTACGCCGTGTGCGCCCTGGCTGCGAT
GGGCCGATTTGTCATCAGACAACGAAAAAATTGCACCTCCGGGCGGGACTCACGCACCAGATACGCTAACT
ATCTTCTGGACACCAAGGGCAAACTATATCGTTGGCGGTCTCCTGTCATCATAGAGAAAGGGGGCAAAATT
GAGGTTGTCTTTCACCTGATCGACCTCAAGAGAGTTGTGCTTGACGGTTCCGCGGCGACTCCTGTACCCTAA
GTTTCAGCGGAACAATGGGGTCGTCCTTAG

>RC05088936.1_montana/PERU/2019

ATGTTGGGGAAATGCTTGACCGCGGGCTGCTGCGCGCGGGGGCTTTTTGGTGGTGGATCGATAAGTTCTG
TTTGGTTTCGCTCGTCAATGCCGAGAACAGCAGCAGCTCCCATTTAAGATAAATCCATATTCGGGCGGTATG
CGGTCTGAATGGCACAAATTGGCTAAATAGAAGGTTTGATTGGGCGGTGTAGACCTTTGTTGTCTTTCCTGT
GTTGACTCATATTGTCTCCTATGGCGCCCTCACCACCAGCCATTTCCTTGACACAGTCGGTTTGATGAAGGTG
TCTGCCGCCGGATATTACCACGGGCAGCGTGTCAAAAGTAGCATTTACGCCGTGTGCGCCCTGGCTGCGAT
GGGCCGATTTGTCATCAGACAACGAAAAAATTGTACCTCCGGGCGGGACTCACGCACCAGATACGCTAACT
ATCTTCTGGACACTAAGGGCAAACTATATCGTTGGCGGTCTCCTGTCATCATAGAGAAAGGGGGTAAAATT
GAGGTTGGAGGTCACCTGATCGACCTCAAGAGAGTTGTGCTTGACGGTTCCGCGGCGACTCCTGTAACCAA
AGTTTCAGCGGAACAATGGGGTCGTCCTTAG

>RC05088935.1_montana/PERU/2019

ATGTTGGGGAAATGCTTGACCGCGGGCTGCTGCTCGCAATTGCTTTTTTTGTGGTGTATCGTGCCGTTCTGT
TTTGTTGCGCTCGTCAACGCCAGCAACAGCAGCAGCTCCCATTTTCAGTTGATTTATAACCTGACGATATGC
GAGCTGAATGGCACAGATTGGCTAAATAGAAGTTTTGATTGGGCGGTAGAGACCTTTGTTATCTTTCCTGTG
TTGACTCATATTGTCTCCTATGGCGCCCTCACCACCAGCCATTTCCTTGACACAGTCGGCCTGATCACCGTGT
CTGCCGCCGGATATTACCACGGGCGGTATGTCTTGAGTAGCATTTATGCCGTCTGCGCCCTGGCTGCGTTAA
CTTGCTTTGTCATCAGGCTAACAAAAAATTGCATGTCCTGGCGTTACTCGTGCACCAGGTACACTAACTATCT
TCTGGACACTAAGGGCAAACTCTATCGTTGGCGGTCTCCTGTCATCATAGAGAAAGGGGGTAAAATTGAGG
TCGAAGGTCACCTGATCGACCTCAAGAGAGTTGTGCTTGACGGTTCCGCGGCAACCCCTGTAACCAAAGTTT
CAGCGGAACAATGGGGTCGTCCTTAG

>RC05088934.1_montana/PERU/2019

ATGTTGGGGAAATGCTTGACCGCGGGCTGCTGCTCGCAATTGCTTTTTTTGTGGTGTATCGTGCCGTTCTGT
TTTGTTGCGCTCGTCAACGCCAGCAACAGCAGCAGCTCCCATTTTCAGTTGATTTATAACCTGACGATATGC
GAGCTGAATGGCACAGATTGGCTAAATAGAAGTTTTGATTGGGCGGTAGAGACCTTTGTTATCTTTCCTGTG
TTGACTCATATTGTCTCCTATGGCGCCCTCACCACCAGCCATTTCCTTGACACAGTCGGCCTGATCACCGTGT
CTGCCGCCGGATATTACCACGGGCGGTATGTCTTGAGTAGCATTTATGCCGTCTGCGCCCTGGCTGCGTTAA
CTTGCTTTGTCATCAGGCTAACAAAAAATTGCATGTCCTGGCGTTACTCGTGCACCAGGTACACTAACTATCT
TCTGGACACTAAGGGCAAACTCTATCGTTGGCGGTCTCCTGTCATCATAGAGAAAGGGGGTAAAATTGAGG
TCGAAGGTCACCTGATCGACCTCAAGAGAGTTGTGCTTGACGGTTCCGCGGCAACCCCTGTAACCAAAGTTT
CAGCGGAACAATGGGGTCGTCCTTAG

>RC05088933.1_montana/PERU/2019

ATGTTGGGGAAATGCTTGACCGCGGGCTGCTGCTCGCAATTGCCTTTTTTGTGGTGTATCGTGCCGTTCTGT
TTTGTTGTGCTCGTCAACGCCAACAACAGCAACAGCTCCCATTTACAGTTGATTTATAACCTGACGATATGTG
AGCTGAATGGCACAGATTGGCTAAATAAAAGTTTTGATTGGGCGGTGGAGACCTTTGTTATCTTTCCTGTGT
TGACTCATATTGTCTCCTACGGCGCCCTCACCACCAGCCATTTCCTTGACACAGTCGGCCTGATCACCGTGTC
TGCCGCCGGATACTACCACGGACGGTATGTCTTAAGTAGCATTTACGCCGTCTGCGCCATGGCTGCGTTAAC
TTGCTTCGTCATCAGGCTAACAAAAAATTGTATGTCCTGGCGTTACTCATGTACCAGGTACACTAATTTTCTT
CTGGACACCAAGGGCAAACTCTATCGTTGGCGGTCTCCTGTCATCATAGAGAAAGGGGGTAAAGTTGAGGT
CGAAGGTCACCTGATCGACCTCAAGAGAGTTGTGCTTGACGGTTCCGCGGCAACCCCTGTAACCAAAGTTT
CAGCGGAACAATGGGGTCGTCCTTAG

>RC05088932.1_montana/PERU/2019

ATGTTGGGGAAATGCTTGACCGCGGGCTGCTGCTCGCAATTGCCTTTTTTGTGGTGTATCGTGCCGTTCTGT
TTTGTTGTGCTCGTCAACGCCAACAACAGCAACAGCTCCCATTTACAGTTGATTTATAACCTGACGATATGTG
AGCTGAATGGCACAGATTGGCTAAATAAAAGTTTTGATTGGGCGGTGGAGACCTTTGTTATCTTTCCTGTGT
TGACTCATATTGTCTCCTACGGCGCCCTCACCACCAGCCATTTCCTTGACACAGTCGGCCTGATCACCGTGTC
TGCCGCCGGATACTACCACGGACGGTATGTCTTAAGTAGCATTTACGCCGTCTGCGCCATGGCTGCGTTAAC
TTGCTTCGTCATCAGGCTAACAAAAAATTGTATGTCCTGGCGTTACTCATGCACCAGATACACTAATTTTCTT
CTGGACACCAAGGGCAAACTCTATCGTTGGCGGTCTCCTGTCATCATAGAGAAAGGGGGTAAAGTTGAGGT
CGAAGGTCACCTGATCGACCTCAAGAGAGTTGTGCTTGACGGTTCCGCGGCAACCCCTGTAACCAAAGTTT
CAGCGGAACAATGGGGTCGTCCTTAG

>RC05088931.1_montana/PERU/2019

ATGTTGGGGAAATGCTTGACCGCGGGCTGCTGCTCGCAATTGCCTTTTTTGTGGTGTATCGTGCCGTTCTGT
TTTGTTGTGCTCGTCAACGCCAACAACAGCAACAGCTCCCATTTACAGTTGATTTATAACCTGACGATATGTG
AGCTGAATGGCACAGATTGGCTAAATAAAAGTTTTGATTGGGCGGTGGAGACCTTTGTTATCTTTCCTGTGT
TGACTCATATTGTCTCCTACGGCGCCCTCACCACCAGCCATTTCCTTGACACAGTCGGCCTGATCACCGTGTC
TGCCGCCGGATACTACCACGGACGGTATGTCTTAAGTAGCATTTACGCCGTCTGCGCCATGGCTGCGTTAAC
TTGCTTCGTCATCAGGCTAACAAAAAATTGTATGTCCTGGCGTTACTCATGTACCAGGTACACTAATTTTCTT
CTGGACACCAAGGGCAAACTCTATCGTTGGCGGTCTCCTGTCATCATAGAGAAAGGGGGTAAAGTTGAGGT
CGAAGGTCACCTGATCGACCTCAAGAGAGTTGTGCTTGACGGTTCCGCGGCAACCCCTGTAACCAAAGTTT
CAGCGGAACAATGGGGTCGTCCTTAG

>RC05088930.1_montana/PERU/2019

ATGTTGGGGAAATGCTTGACCGCGGGCTGCTGCTCGCAATTGCCTTTTTTGTGGTGTATCGTGCCGTTCTGT
TTTGTTGTGCTCGTCAACGCCAACAACAGCAACAGCTCCCATTTACAGTTGATTTATAACCTGACGATATGTG
AGCTGAATGGCACAGATTGGCTAAATGAAAGTTTTGATTGGGCGGTGGAGACCTTTGTTATCTTTCCTGTGT
TGACTCATATTGTCTCCTACGGCGCCCTCACCACCAGCCATTTCCTTGACACAGTCGGCCTGATCACCGTGTC
TGCCGCCGGATACTACCACGGACGGTATGTCTTAAGTAGCATTTACGCCGTCTGCGCCATGGCTGCGTTAAC
TTGCTTCGTCATCAGGCTAACAAAAAATTGTATGTCCTGGCGTTACTCATGTACCAGGTACACTAATTTTCTT
CTGGACACCAAGGGCAAACTCTATCGTTGGCGGTCTCCTGTCATCATAGAGAAAGGGGGTAAAGTTGAGGT
CGAAGGTCACCTGATCGACCTCAACAGAGTTGTGCTTGACGGTTCCGCGGCAACCCCTGTAACCAAAGTTTC
AGCGGAACAATGGGGTCGTCCTTAG

>RC05088929.1_montana/PERU/2019

ATGTTGGGGAAATGCTTGACCGCGGGCTGCTGCTCGCAATTGCTTTTTTTGTGGTGTATCGTGCCGTTCTGT
TTTGTTGCGCTCGTCAACGCCAGCAACAGCAGCAGCTCCCATTTTCAGTTGATTTATAACCTGACGATATGC
GAGCTGAATGGCACAGATTGGCTAAATAGAAATTTTGATTGGGCGGTAGAGACCTTTGTTATCTTTCCTGTG
TTGACTCATATTGTCTCCTATGGCGCCCTCACCACCAGCCATTTCCTTGACACAGTCGGCCTGATCACCGTGT
CTGCCGCCGGATATTACCACGGGCGGTATGTCTTGAGTAGCATTTATGCCGTCTGCGCCCTGGCTGCATTAA
TTTGCTTTGTCATCAGGCTAACAAAAAATTGCATGTCCTGGCGTTACTCGTGCACCAGGTACACTAACTATCT
TCTGGACACTAAGGGCAAACTCTATCGTTGGCGGTCTCCTGTCATCATAGAGAAAGGGGGTAAAATTGAGG
TCGAAGGTCACCTGATCGACCTCAAGAGAGTTGTGCTTGACGGTTCCGCGGCAACCCCTGTAACCAAAGTTT
CAGCGGAACAATGGGGTCGTCCTTAG

>RC05088928.1_montana/PERU/2019

ATGTTGGGGAAATGCTTGACCGCGGGCTGCTGCTCGCAATTGCTTTTTTTGTGGTGTATCGTGCCGTTCTGT
TTTGTTGCGCTCGTCAACGCCAGCAACAGCAGCAGCTCCCATTTTCAGTTGATTTATAACCTGACGATATGC
GAGCTGAATGGCACAGATTGGCTAAATAGAAGTTTTGATTGGGCGGTAGAGACCTTTGTTATCTTTCCTGTG
TTGACTCATATTGTCTCCTATGGCGCCCTCACCACCAGCCATTTCCTTGACACAGTCGGCCTGATCACCGTGT
CTGCCGCCGGATATTACCACGGGCGGTATGTCTTGAGTAGCATTTATGCCGTCTGCGCCCTGGCTGCGTTAA
CTTGCTTTGTCATCAGGCTAACAAAAAATTGCATGTCCTGGCGTTACTCGTGCACCAGGTACACTAACTATCT
TCTGGACACTAAGGGCAAACTCTATCGTTGGCGGTCTCCTGTCATCATAGAGAAAGGGGGTAAAATTGAGG
TCGAAGGTCACCTGATCGACCTCAAGAGAGTTGTGCTTGACGGTTCCGCGGCAACCCCTGTAACCAAAGTTT
CAGCGGAACAATGGGGTCGTCCTTAG

>RC05088927.1_montana/PERU/2019

ATGTTGGGGAAATGCTTGACCGCGGGCTGCTGCTCGCAATTGCTTTTTTTGTGGTGTATCGTGCCGTTCTGT
TTTGTTGCGCTCGTCAACGCCAGCAACAGCAGCAGCTCCCATTTTCAGTTGATTTATAACCTGACGATATGC
GAGCTGAATGGCACAGATTGGCTAAATAGAAGTTTTGATTGGGCGGTAGAGACCTTTGTTATCTTTCCTGTG
TTGACTCATATTGTCTCCTATGGCGCCCTCACCACCAGCCATTTCCTTGACACAGTCGGCCTGATCACCGTGT
CTGCCGCCGGATATTACCACGGGCGGTATGTCTTGAGTAGCATTTATGCCGTCTGCGCCCTGGCTGCGTTAA
CTTGCTTTGTCATCAGGCTAACAAAAAATTGCATGTCCTGGCGTTACTCGTGCACCAGGTACACTAACTATCT
TCTGGACACTAAGGGCAAACTCTATCGTTGGCGGTCTCCTGTCATCATAGAGAAAGGGGGTAAAATTGAGG
TCGAAGGTCACCTGATCGACCTCAAGAGAGTTGTGCTTGACGGTTCCGCGGCAACCCCTGTAACCAAAGTTT
CAGCGGAACAATGGGGTCGTCCTTAG

>RC05088926.1_montana/PERU/2019

ATGTTGGGGAAATGCTTGACCGCGGGCTGCTGCTCGCAATTGCTTTTTTTGTGGTGTATCGTGCCGTTCTGT
TTTGTTGCGCTCGTCAACGCCAGCAACAGCAGCAGCTCCCATTTTCAGTTGATTTATAACCTGACGATATGC
GAGCTGAATGGCACAGATTGGCTAAATAGAAGTTTTGATTGGGCGGTAGAGACCTTTGTTATCTTTCCTGTG
TTGACTCATATTGTCTCCTATGGCGCCCTCACCACCAGCCATTTCCTTGACACAGTCGGCCTGATCACCGTGT
CTGCCGCCGGATATTACCACGGGCGGTATGTCTTGAGTAGCATTTATGCCGTCTGCGCCCTGGCTGCGCTAA
CTTGCTTTGTCATCAGGCTAACAAAAAATTGCATGTCCTGGCGTTACTCGTGCACCAGGTACACTAACTATCT
TCTGGACACTAAGGGCAAACTCTATCGTTGGCGGTCTCCTGTCATCATAGAGAAAGGGGGTAAAATTGAGG
TCGAAGGTCACCTGATCGACCTCAAGAGAGTTGTGCTTGACGGTTCCGCGGCAACCCCTGTAACCAAAGTTT
CAGCGGAACAATGGGGTCGTCCTTAG

>RC05088925.1_montana/PERU/2019

ATGTTGGGGAAATGCTTGACCGCGGGCTGCTGCTCGCAATTGCTTTTTTTGTGGTGTATCGTGCCGTTCTGT
TTTGTTGCGCTCGTCAACGCCAGCAACAGCAGCAGCTCCCATTTTCAGTTGATTTATAACCTGACGATATGC
GAGCTGAATGGCACAGATTGGCTAAATAGAAGTTTTGATTGGGCGGTAGAGACCTTTGTTATCTTTCCTGTG
TTGACTCATATTGTCTCCTATGGCGCCCTCACCACCAGCCATTTCCTTGACACAGTCGGCCTGATCACCGTGT
CTGCCGCCGGATATTACCACGGGCGGTATGTCTTGAGTAGCATTTATGCCGTCTGCGCCCTGGCTGCGTTAA
CTTGCTTTGTCATCAGGCTAACAAAAAATTGCATGTCCTGGCGTTACTCGTGCACCAGGTACACTAACTATCT
TCTGGACACTAAGGGCAAACTCTATCGTTGGCGGTCTCCTGTCATCATAGAGAAAGGGGGTAAAATTGAGG
TCGAAGGTCACCTGATCGACCTCAAGAGAGTTGTGCTTGACGGTTCCGCGGCAACCCCTGTAACCAAAGTTT
CAGCGGAACAATGGGGTCGTCCTTAG

>MH791391.1_Sublineage_1A(PERU-2017/7)

ATGTTGGGGAAATGCTTGACCGCGGGCTGCTGCTCGCAATTGCTTTTTTTGTGGTGTATCGTGCCGTTCTGT
TTTGTTGCGCTCGTCAACGCCAACAACAGCAGCAGCTCCCATTTACAGTTGATTTATAACCTGACGGTATGT
GAGCTGAATGGCACAGATTGGCTAAATAGAAGGTTTGATTGGGCGGTGGAGACCTTTGTTATCTTCCCTGT
GTTGACTCATATTGTCTCCTATGGCGCCCTCACCACCAGCCATTTCCTTGACACAGTCGGCCTGATCACCGTG
TCTGCCGCTGGATATTACCACGGGCGGTATGTCTTGAGTAGCATTTACGCCGTCTGCGCCCTGGCTGCGTTA
ACTTGCTTCGTCATCAGGCTAACAAAAAATTGTATGTCCTGGCGTTACTCATGCACCAGGTACACCAACTATC
TTCTGGACACCAAGGGCAAACTCTATCGTTGGCGGTCTCCTGTCATCATAGAGAAAGGGGGTAAAATTGAG
GTCGAAGGTCACCTGATCGACCTCAAGAGAGTTGTGCTTGACGGTTCCGCGGCAACCCCTGTAACCAAAGT
TTCAGCGGAACAATGGGGTCGTCCTTAG

>MH791390.1_Sublineage_1A(PERU-2017/6)

ATGTTGGGGAAATGCTTGACCGCGGGCTGCTGCTCGCAATTGCCTTTTTTGTGGTGTATCGTGCCGTTCTGT
TTTGTTGCGCTCGTCAACGCCAACAACAGCAGCAGCTCCCATTTACAGTTGATTTATAACCTGACGATATGT
GAGCTGAATGGCACAGATTGGCTAAATAAAAGTTTTGATTGGGCGGTGGAGACCTTTGTTATCTTTCCTGTG
TTGACTCATATTGTCTCCTATGGCGCCCTCACCACCAGCCATTTCCTTGACACAGTCGGCCTGATCACCGTGT
CTGCCGCCGGATATTACCACGGGCGGTATGTCTTGAGTAGCATTTACGCCGTCTGCGCCCTGGCTGCGTTAA
TTTGCTTCGTCATCAGGCTAACAAAAAATTGTATGTCCTGGCGTTACTCATGCACCAGGTACACCAACTTTCT
TCTGGACACCAAGGGCAAACTCTATCGTTGGCGGTCTCCTGTCATCATAGAGAAAGGGGGTAAAATTGAGG
TCGAAGGTCACCTGATCGACCTCAAGAGAGTTGTGCTTGACGGTTCCGCGGCAACCCCTGTAACCAAAGTTT
CAGCGGAACAATGGGGTCGTCCTTAG

>MH791388.1_Sublineage_1A(PERU-2016/16)

ATGTTGGGGAAATGCTTGACCGCGGGCTGCTGCTCGCAATTGCTTTTTTTGTGGTGTATCGTGCCGTTCTGT
TTTGTTGCGCTCGTCAACGCCAACAACAGCAGCAGCTCCCATTTACAGTTGATTTATAACCTGACGATATGT
GAGCTGAATGGCACAGATTGGCTAAATAAAAGTTTTGATTGGGCGGTGGAGACCTTTGTTATCTTTCCTGTG
TTGACTCATATTGTCTCCTATGGCGCCCTCACCACCAGCCATTTCCTTGACACAGTCGGCCTGATCACCGTGT
CTGCCGCCGGATATTACCACGGGCGGTATGTCTTGAGTAGCATTTACGCCGTCTGCGCCCTAGCTGCGTTAA
CTTGCTTCGTCATCAGGCTAACGAAAAATTGTATGTCCTGGCGTTACTCATGCACCAGATACACTAACTTTCT
TCTGGACACCAAGGGCAAACTCTATCGTTGGCGGTCTCCTGTCATCATAGAGAAAGGGGGTAAAATTGAGG
TCGAAGGTCACCTGATCGACCTCAAGAGAGTTGTGCTTGACGGTTCCGCGGCAACCCCTGTAACCAAAGTTT
CAGCGGAACAATGGGGTCGTCCTTAG

>MH791386.1_Sublineage_1A(PERU-2016/5)

ATGTTGGGGAAATGCTTGACCGCGGGCTGCTGCTCGCAATTGCCTTTTTTGTGGTGTATCGTGCCGTTCTGT
TTTGTTGTGCTCGTCAACGCCAACAACAGCAGCAGCTCCCATTTACAGTTGATTTATAACCTGACGATATGTG
AGCTGAATGGCACAGATTGGCTAAATAAAAGTTTTGATTGGGCGGTGGAGACCTTTGTCATCTTTCCTGTGT
TGACTCACATTGTCTCCTATGGCGCCCTCACCACCAGCCATTTCCTTGACACAGTCGGCCTGATCACCGTGTC
TGCCGCCGGATATTACCACGGGCGGTATGTCTTGAGTAGCATTTACGCCGTCTGCGCCCTGGCTGCGTTAAC
TTGCTTCGTTATCAGGCTAACAAAAAATTGTATGTCCTGGCGTTACTCATGCACCAGATACACTAACTTTCTT
CTGGACACCAAGGGCAAACTCTATCGTTGGCGGTCTCCTGTCATCATAGAGAAAGGGGGCAAAATTGAGGT
CGAAGGTCACCTGATCGACCTCAAGAGAGTTGTGCTTGACGGTTCCGCGGCAACCCCTGTAACCAAAGTTT
CAGCGGAACAATGGGGTCGTCCTTAG

>MH791385.1_Sublineage_1A(PERU-2016/4)

ATGTTGGGGAAATGCTTGACCGCGGGCTGCTGCTCGCAATTGCCTTTTTTGTGGTGTATCGTGCCGTTCTGT
TTTGTTGCGCTCGTCAACGCCAACAACAGCAGCAGCTCCCATTTACAGTTGATTTATAACCTGACGATATGT
GAGCTGAATGGCACAGATTGGCTAAATAAAAGTTTTGATTGGGCGGTGGAGACCTTTGTTATTTTTCCTGTG
TTGACTCATATTGTCTCCTATGGCGCCCTCACCACCAGCCATTTCCTTGACACAGTCGGCCTGATCACCGTGT
CTGCCGCCGGATATTACCACGGGCGGTATGTCTTGAGTAGCATTTACGCCGTCTGCGCCCTGGCTGCGTTAA
CTTGCTTCGTCATCAGGCTAACAAAAAACTGTATGTCCTGGCGTTACTCATGCACCAGATACACTAACTTTCT
TCTGGACACCAAGGGCAAGCTCTATCGTTGGCGGTCTCCTGTCATCATAGAGAAAGGGGGTAAAATTGAGG
TCGAAGGTCACCTGATCGACCTCAAGAGAGTTGTGCTTGACGGTTCCGCGGCAACCCCTGTAACCAAAGTTT
CAGCGGAACAATGGGGTCGTCCTTAG

>MH791383.1_Sublineage_1A(PERU-2016/2)

ATGTTGGGGAAATGCTTGACCGCGGGCTGCTGCTCGCAATTGCCTTTTTTGTGGTGTATCGTGCCGTTCTGT
TTTGTTGCGCTCGTCAACGCCAACAACAGCAGCAGCTCCCATTTACAGTTGATTTATAACCTGACGATATGT
GAGCTGAATGGCACAGATTGGCTAAATAAAAGTTTTGATTGGGCGGTGGAGACCTTTGTTATCTTTCCTGTG
TTGACTCATATTGTCTCCTATGGCGCCCTCACTACCAGCCATTTCCTTGACACAGTCGGCCTGATCACCGTGT
CTGCCGCCGGATACTACCACGGGCGGTATGTCTTGAGTAGCATTTACGCCGTCTGCGCCCTGGCTGCGTTAA
CTTGCTTTGTCATCAGGCTAACAAAAAATTGTATGTCCTGGCGTTACTCATGCACCAGATACACTAACTTTCT
TCTGGACACCAAGGGCAAACTCTATCGTTGGCGGTCTCCTGTCATCATAGAGAAAGGGGGTAAAATTGAGG
TCGAAGGTCACCTGATCGACCTCAAGAGAGTTGTGCTTGACGGTTCCGCGGCAACCCCTGTAACCAAAGTTT
CAGCGGAACAATGGGGTCGTCCTTAG

>MH791379.1_Sublineage_1A(PERU-2015/17)

ATGTTGGGGAAATGCTTGACCGCGGGCTGCTGCTCGCAATTGCCTTTTTTGTGGTGTATCGTGCCGTTCTGT
TTTGTTGCGCTCGTCAACGCCAACAACAGCAGCAGCTCTCATTTACAGTTGATTTATAACCTGACGATATGTG
AGCTGAATGGCACAGATTGGCTAAATAAAAGTTTTGATTGGGCGGTGGAGACCTTTGTTATCTTTCCTGTGT
TGACTCATATTGTCTCCTATGGCGCCCTCACCACCAGCCATTTCCTTGACACAGTCGGCCTGATCACCGTGTC
TGCCGCCGGATACTACCACGGGCGGTATGTCTTGAGTAGCATTTACGCCGTCTGCGCCCTGGCTGCGTTAAC
TTGCTTCGTCATCAGGCTAACAAAAAATTGTATGTCCTGGCGTTACTCATGCACCAGATACACTAACTTTCTT
CTGGACACCAAGGGCAAACTCTATCGTTGGCGGTCTCCTGTCATCATAGAGAAAGGGGGTAAAATTGAGGT
CGAAGGTCACCTGATCGACCTCAAGAGAGTTGTGCTTGACGGTTCCGCGGCAACCCCTGTAACCAAAGTTT
CAGCGGAACAATGGGGTCGTCCTTAG

>MH791378.1_Sublineage_1A(PERU-2015/15)

ATGTTGGGGAAATGCTTGACCGCGGGCTGCTGCTCGCAATTGCTTTTTTTGTGGTGTATCGTGCCGTTCTGT
TTTGTTGCGCTCGTCAACGCCAACAACAGCAGCAGCTCCCATTTACAGTTGATTTATAACCTGACGATATGT
GAGCTGAATGGCACAGATTGGCTAAATAAAAGTTTTGATTGGGCGGTGGAGACCTTCGTTATCTTTCCTGTG
TTGACTCATATTGTCTCCTATGGCGCCCTCACCACCAGCCATTTCCTTGACACAGTCGGCCTGATCACCGTGT
CTGCCGCCGGATATTACCACAGGCGGTATGTCTTGAGTAGCATTTACGCCGTCTGCGCCCTGGCTGCGTTAA
CTTGCTTCGTCATCAGGCTAACAAAAAATTGTATGTCCTGGCGTTACTCATGCACCAGGTACACTAACTTTCT
TCTGGACACCAAGGGCAAACTCTATCGTTGGCGGTCTCCTGTCATCATAGAGAAAGGGGGTAAAATTGAGG
TCGAAGGTCACCTGATCGACCTCAAGAGAGTTGTGCTTGACGGTTCCGCGGCAACCCCTGTAACCAAAGTTT
CAGCGGAACAATGGGGTCGTCCTTAG

>MH791376.1_Sublineage 1A(PERU-2015/13)

ATGTTGGGGAAATGCTTGACCGCGGGCTGCTGCTCGCAATTGCTTTTTTTGTGGTGTATCGTGCCGTTCTGT
TTTGTTGCGCTCGTCAACGCCAACAACAGCAGCAGCTCCCATTTACAGTTGATTTATAACCTGACGATATGT
GAGCTGAATGGCACAGATTGGCTAAATAAAAGTTTTGATTGGGCGGTGGAGACCTTTGTTATCTTTCCTGTG
TTGACTCATATTGTCTCCTATGGCGCCCTCACCACCAGCCATTTCCTTGACACAGTCGGCCTGATCACCGTGT
CTGCCGCCGGATATTACCACAGGCGGTATGTCTTGAGTAGCATTTACGCCGTCTGCGCCCTGGCTGCGTTAA
CTTGCTTCGTCATCAGGCTAACAAAAAATTGTATGTCCTGGCGTTACTCATGCACCAGGTACACTAACTATCT
TCTGGACACCAAGGGCAAACTCTATCGTTGGCGGTCTCCTGTCATCATAGAGAAAAGGGGTAAAATTGAGG
TCGAAGGTCACCTGATCGACCTCAAGAGAGTTGTGCTTGACGGTTCCGCGGCAACCCCTGTAACCAAAGTTT
CAGCGGAACAATGGGGTCGTCCTTAG

>MF326985.1_Sublineage_1A(NADC34)

ATGTTGGGGAAATGCTTGACCGCGGGCTGCTGCTCGCAATTGCCTTTTTTGTGGTGTATCGTGCCGTTCTGT
TTTGTTGCGCTCGTCAACGCCAACAACAGCAGCAGCTCCCATTTACAGTTGATTTATAACCTGACGATATGT
GAGCTGAATGGCACAGATTGGCTAAATAAAAGTTTTGATTGGGCGGTGGAGACCTTTGTTATTTTTCCTGTG
TTGACTCATATTGTCTCCTATGGCGCCCTCACCACCAGCCATTTCCTTGACACAGTCGGCCTGATCACCGTGT
CTGCCGCCGGATATTACCACGGGCGGTATGTCTTGAGTAGCATTTACGCCGTCTGCGCCCTGGCTGCGTTAA
CTTGCTTCGTCATCAGGCTAACAAAAAATTGTATGTCCTGGCGTTACTCATGCACCAGATATACTAACTTTCT
TCTGGACACCAAGGGCAAACTCTATCGTTGGCGGTCTCCTGTCATCATAGAGAAAGGGGGTAAAATTGAGG
TCGAAGGTCACCTGATCGACCTCAAGAGAGTTGTGCTTGACGGTTCCGCGGCAACCCCTGTAACCAAAGTTT
CAGCGGAACAATGGGGTCGTCCTTAG

>MF279253.1_Sublineage_8D(DMZC181)

ATGTTGGGGAATTGCTTGACAGCTGTTTGTTGCTCGCGATTGTTTTCTTTGTGGTATATCGTGCCGTTTTGTT
TTGCTGTGCTCGTCAACGCCAACARCAGCAACAGCTCTCATTTTCAGTTGATTTATAACTTGACGATATGCGA
GCTGAATGGCACAGACTGGCTGAGCCACAACTTTGATTGGGCCGTGGAGACTTTTGTCATTTTTCCCGTGCT
AACTCACATCGTTTCCTATGGTGCACTCACCACCAGCCACTTCCTCGACACGGTTGGTCTGGTTACAGTGTCC
ACCGCCGGGTATTACCACGGGCGGTATGTCTTGAGCAGCATCTATGCAATCTGTGCCTTAGCAGCATTGACT
TGCTTCTTCATTAGGCTTACGAAAAATTGCATGTCCTGGCGTTACTCTTGTACCAGATATACCAACTTCCTTCT
AGACACTAAGGGCAGGCTCTATCGTTGGCGGTCTCCCGTCATCATAGAGAAAGGGGGTAAGGTTGAGGTT
GAAGGTCACCTAATCGACCTCAAAAGAGTCGTGCTTGATGGTTCCGCGGCAACYCCTTTAACCAAAGTTTCA
GCGGAGCAATGGGGTCGTCCCTAG

>KF611905.1_Sublineage_1C(HENAN-XINX)

ATGTTGGTGAAATGCTTGGCCGCGGGTTGTTGCTCGCAATTGCCTTTTTTGTGGTGTATCGTGCCGTTCTATT
TTGCTGCGCTCGTCAACGCCAACAGCAACAGCAGCTCCCATCTACAGTTGATTTATAACCTGACGATATGTG
AGCTGAATGGTACGGATTGGCTGGGATCAAATTTTGATTGGGCAGTGGAGACTTTCGTCATCTTTCCTGTAT
TGACTCATATTGTCTCTTACGGCGCCCTTACCACTAGCCATTTTCTTGACACGGTCGGCCTGATCACTGTGTC
CACCGCCGGATATTTTCACAAGCGGTATGTGTTGAGTAGCATCTACGCTGTCTGTGCCCTGGCTGCGTTGGT
TTGCTTCGCCATTAGGTTGGCAAAAAATTGCATGTCCTGGCGCTACTCATGCACCAGATATACCAATTTTCTT
CTGGACACTAAGGGCAAACTCTACCGCTGGCGGTCACCCGTCATCATAGAGAAGGAGGGTAAAGTTGATG
TGGGTGGTCACTTAATCGACCTCAAGAGAGTTGTGCTTGATGGTTCCGCGGCAACCCCTGTAACCAAGATTT
CAGCGGAACAATGGGGTCGTCCATAG

>JX512910.2_Sublineage_8E(EE.UU-SRV07)

ATGTTGGGGAAGTGCTTGACCGCGTGCTGTTGCTCGCGATTGCTTTTTTTGTGGTGTATCGTGCCGTTCTATC
TTGCTGTGCTCGTCAACGCCAGCAACAACAACAGCCCTCATATTCAGTTGATTTATAACTTAACGCTATGTGA
GCTGAATGGCACAGATTGGCTGGCACAAAAATTTGACTGGGCAGTGGAGACTTTTGTCATCTTCCCCGTGTT
GACTCACATTGTTTCCTATGGGGCACTCACCACCAGCCATTTCCTTGACACAGTTGGTCTGGCCACTGTGTCC
ACCGCCGGATATTATCACGGGCGGTATGTCTTGAGTAGCATTTACGCAGTCTGTGCTCTGGCTGCGCTGATT
TGCTTTGTCATTAGGCTTGCGAAGAACTGCATGTCCTGGCGCTACTCTTGTACCAGATATACCAACTTCCTTC
TGGACACTAAGGGCAGACTCTATCGTTGGCGGTCGCCCGTCATTGTGGAGAAAGGGGGTAAGGTTGAGGT
CGAAGGTCACCTCATCGACCTCAAAAGAGTTGTGCTTGATGGTTCCGCGGCAACCCCTTTAACCAGAGTTTC
AGCGGAACAATGGGGTCGTCTCTAG

>JN660150.1_Sublineage_1B(NADC31)

ATGTTGGGGAAATGCTCGACCGCGGGCTGCTGCTCGCCATTGCTTTTTTTGTGGTGTATCGTGCCGTCCTGT
TTAGTTGCGCTCGTCAACGCCAGCAAAAACAACAGCTCCCATTTACAGTCGATTTATAACCTGACGATATGT
GAGCTGAATGGCACAGATTGGCTAAATAAAAAATTTGACTGGGCAGTGGAGACCTTTGTCATTTTTCCTGTA
TTGACTCATATCGTCTCCTATGGTGCCCTCACCACCAGCCATTTCCTTGACGCAGTCGGTTTGGTCATCGTGT
CCACCGCCGGATACTATCACGGGCGGTATGTCCTGAGCAGCATTTACGCTGTCTGCGCCTTGGCCGCGCTG
ATTTGCTTCGCCATCAGGTTAACGAAAAACTGCATGTCCTGGCGCTACTCATGTACTAGGTATACTAACTTTC
TTCTAGACACCAAGGGCAAACTCTATCGTTGGCGGTCTCCCGTCATCATAGAAAAAAAAGGGAAAATCGAG
GTCAACGGTCACTTGATCGACCTCAAGAGAGTTGTGCTTGATGGTTCCGCAGCAACCCCTGTAACCAAAGTT
TCAGCGGAACAATGGGGTCATCCTTAG

>EU807840.1_Sublineage_9D(CHINA-CH-1R)

ATGTTGGGGAAATACTTGACCACGGGCTGCTGCTCGCGATTGCTTTCTTTGTGGTGTATCGTGCCGTTCTGT
TTTGCTGTGCTCGTCAACGCCAACAGCAACAGCAGCTCTCAATTTCAGTTGATTTATAACTTGACGCTATGTG
AGCTGAATGGCACAGATTGGCTGGCTAACAAATTTGACTGGGCAGTGGAGACTTTTGTCATCTTTCCCGTGT
TGACTCACATTGTGTCCTATGGGGCACTCACCACCAGCCATTTCCTTGACACAGTTGGTCTGGTCACTGTGTC
CACCGCCGGGTTTTATCACGGGCGGTATGTCTTGAGTAGCATCTACGCGGTCTGTGCTCTGGCTGCGTTGAT
TTGCTTCGTCATTAGGCTTGCGAAGAACTGCATGTCCTGGCGCTACTCTTGTACCAGATATACCAACTTCCTT
CAGGACACTAAGGGCAGACTCTATCGTTGGCGGTCGCCCGTTATTGTAGAGAAAGGGGGTAAGGTTGAGG
TCGAGGGTCACCTGATCGACCTCAAAAGAGTTGTGCTTGATGGTTCCGTGGCAACCCCTTTAACCAGAGTTT
CAGCGGAACAATGGGGTCGTCTCTAG

>EF536003.1_Sublineage_5A(VR2332)

ATGTTGGAGAAATGCTTGACCGCGGGCTGTTACTCGCAATTGCTTTCTTTGTGGTGTATCGTGCCGTTCTGTT
TTGCTGTGCTCGTCAACGCCAGCAACGACAGCAGCTCCCATCTACAGCTGATTTACAACTTGACGCTATGTG
AGCTGAATGGCACAGATTGGCTAGCTAACAAATTTGATTGGGCAGTGGAGAGTTTTGTCATCTTTCCCGTTT
TGACTCACATTGTCTCCTATGGTGCCCTCACTACTAGCCATTTCCTTGACACAGTCGCTTTAGTCACTGTGTCT
ACCGCCGGGTTTGTTCACGGGCGGTATGTCCTAAGTAGCATCTACGCGGTCTGTGCCCTGGCTGCGTTGACT
TGCTTCGTCATTAGGTTTGCAAAGAATTGCATGTCCTGGCGCTACGCGTGTACCAGATATACCAACTTTCTTC
TGGACACTAAGGGCAGACTCTATCGTTGGCGGTCGCCTGTCATCATAGAGAAAAGGGGCAAAGTTGAGGT
CGAAGGTCATCTGATCGACCTCAAAAGAGTTGTGCTTGATGGTTCCGTGGCAACCCCTATAACCAGAGTTTC
AGCGGAACAATGGGGTCGTCCTTAG

>DQ988080.1_Sublineage_8A(Ingelvac_ATP)

ATGTTGGGGAGATGCTTGACCGCGGGCTGTTGCTCGCGATTGCTTTCTTTGTGGTGTATCGTGCCATTTTGT
TTTGCTGCGCTCGTCAACGCCAACAGCAACAGCAGCTCTCATCTTCAGTTAATTTACAACTTGACGCTATGTG
AGCTGAATGGCACAGATTGGCTGAAAGACAAATTTGATTGGGCATTGGAGACTTTTGTCATCTTTCCCGTGT
TGACTCACATTGTCTCATATAGTGCACTCACCACTAGCCATTTCCTTGACACAGTCGGTCTGGTTACTGTGTC
TACTGCCGGGTTCTACCACGGGCGGTATGTTCTGAGTAGCATCTACGCGGTCTGCGCTCTGGCCGCATTGAC
TTGCTTCGTCATTAGGCTTGCGAAGAACTGCATGTCCTGGCGCTACTCTTGTACCAGATATACTAACTTCCTT
CTGGACACTAAGGGCAGACTCTATCGCTGGCGGTCGCCCGTTATCATAGAGAAAGGGGGTAAGGTTGAGG
TCGAAGGTCACCTGATCGACCTCAAAAGAGTTGTGCTTGATGGTTCCGTGGCAACCCCTTTAACCAGAGTTT
CAGCGGAACAATGGGGTCGTCTTTAG

>DQ473474.1_Sublineage_5B(COREA_DEL_SUR-LMY)

ATGTTGGGGAAATGCTTGACCGCGGGCTGTTGCTCGCAATTGCTTTTTTTGTGGTGTATCGTGCCGTCTTGT
TTTGTTGCGATCGTCAGCGCCAACAACAGCAGCAGCTCAAATTTACAGCTGATTTACAACTTGACGCTATGT
GAGCTGAATGGCACAGATTGGCTAGCTAACAGATTTGACTGGGCGGTGGAGTGTTTTGTTATTTTTCCTGTA
TTGACTCACATTGTCTCTTATGGTGCCCTAACCACTAGCCACTTCCTTGACACAGTCGGTTTGGTCACTGTGT
CTACCGCCGGATTTGTTCACGGGCGGTATGTTTTGAGTAGCATTTACGCGGTCTGTGCCCTGGCTGCGTTGA
TTTGCTTCGTCATTAGGCTTGCGAAGAATTGCATGTCCTGGCGCTACTCATGTACCAGATATACCAACTTTCT
TCTAGATACCAAGGGCAGACTCTACCGTTGGCGGTCGCCTGTCATTATAGAGAAAAGGGGCAAAGTTGAG
GTCGAGGGTCAACTAATCGACCCCAAAAGAGTTGTGCTTGATGGTTCCCCGGCAACCCCTGTAACCAGAGT
TTCAGCGGAACAATGGGGTCATCCTTAG

>DQ306879.1_Sublineage_1E(EE.UU-99-3584)

ATGTTGGGGAAATGCTTGACCGCGGGCTGTTGCTCGCAATTGCCTTTTTTGTGGTGTATCGTGCCGTTCTGT
TTTGCTGCGCTCGTCAACGCCGGCAGCAACAGCAGCTCCCATTTACAGTTGATTTATAACCTGACGATATGT
GAGCTGAATGGCACAGATTGGCTGAACGACAAATTTGATTGGGCGGTGGAGACTTTTGTCATCTTTCCTGT
GTTGACTCACATTGTTTCMTATGGCGCCCTCACCACCAGCCATTTCCTTGACACAGTCGGTCTAGTTACTGTG
TCTACCGCCGGATATTACCATAGGCGGTATGTATTGAGTAGCATTTACGCTGTCTGTGCCCTGGCTGCGTTG
ATTTGCTTCGTCATCAGGTTGACGAAGAATTGCATGTCCTGGCGCTACTCATGCACCAGATATACTAACTTTC
TTTTGGACACCAAGGGCAGACTCTATCGTTGGCGGTCACCCGTCATCATAGAGAAAGGGGGCAAAGTTGA
GGTTGAAGGTCACCTGATCGACCTCAAGAGAGTTGTGCTTGACGGTTCCGCGGCAACCCCCGTAACCAAAG
TTTCAGCAGAACGATGGGGTCGTCCCTAG

>DQ176019.1_Sublineage_1F(EE.UU-MN184A)

ATGTTGGGGAAATGCTTGACCGCGGGCTATTGCTCGCAATTGCTTTTTTTGTGGTGTATCGTGCCGTTCTGT
CTTGCTGCGCTCGTCAACGCCGACAGCAACAGCAGCTCCCATTTACAGTTGATTTATAAMTTAACGATATGT
GAGCTGAATGGCACAGACTGGCTGAACAATCATTTTAGTTGGGCAGTGGAGACTTTCGTTATCTTTCCTGTG
TTGACTCATATTGTTTCCTACGGCGCCCTCACTACCAGCCACCTCCTTGACACGGTCGGCCTGATCACTGTGT
CCACCGCCGGATACTGCCATAAGCGGTATGTCTTGAGTAGCATCTATGCTGTCTGCGCCCTGGCTGCGCTGA
TTTGCTTCGTCATCAGGTTGACGAAAAATTGTATGTCCTGGCGCTACTCATGTACCAGATATACCAACTTTCT
TCTGGACACCAAGGGCAGACTCTATCGCTGGCGGTCACCCGTCATCATAGAGAAAAGGGGTAAAATTGAG
GTTGGAGGTGACCTGATCGACCTCAAGAGAGTTGTGCTTGATGGTTCCGCGGCAACCCCTGTAACCAAAGT
TTCAGCGGAACAATGGGGTCGTCCTTAG

>AF325691.1_Sublineage_8B_(EE.UU-NVSL_97-7985_IA_1-4-2)

ATGTTGGAGAAATGCTTGACCGCGGGCTGTTGCTTGCGATTGCCTTCTTTGTGGTGTATCGTGCCGTTCTGT
TTTGCTGTGCTCGTCAACGCCAACAACAGCAGCAGCTCCCATTTTCAGTCGATTTATAACTTAACGCTATGTG
AGCTGAATGGCACAGAATGGCTGAGTGAGAAATTTGATTGGGCAGTGGAGACTTTTGTCATCTTTCCCGTG
TTAACTCACATTGTTTCCTATGGTGCACTCACCACCAGCCATTTCCTTGACACAGTTGGTCTGGTTACTGTGTC
CACCGCCGGGTTTCTCCACAGGCGGTATGTCTTGAGCAGCGTCTACGCGGTCTGTGCTCTGGCTGCGTTGAT
TTGCTTCATCATTAGGCTTGCGAAGAACTGCATGTCCTGGCGCTACTCTTGTACCAGATATACCAACTTTCTT
CTGGACACTAAGGGCAAACTCTATCGTTGGCGGTCGCCCGTTATCATAGAGAAAGGGGGCAGGGTTGAGG
TCGAAGGTCACCTGATCGACCTCAAAAGAGTTGTGCTTGATGGTTCCGCGGCAACCCCTTTAACCAGAGTTT
CAGCGGAACAATGGGGTCGTCTCTAG

>AF184212.1_lineage_7(CHINA-SP)

ATGTTGGGGAAATGCTTGACCGCGGGTTGCTGCTCGCGATTGCTTTCTTTTTGGTGTATCGTGCCGTTCTGTT
TTGCTGTGCTCGTCAACGCCAGCTACAGCAGCAGCTCTCATTTACAGTTGATTTATAACTTGACGCTATGTGA
GCTGAATGGTACAGATTGGCTGGCTAATAAATTTGATTGGGCAGTGGAGAGTTTTGTCATCTTTCCTGTGTT
GACCCACATCGTTTCCTATGGTGCACTAACCACCAGCCACTTCCTTGACACAGTTGGTCTGGTTACTGTGTCT
ACCGCCGGGTTTTATCATGGGCGGTATGTCCTGAGTAGCATCTACGCGGTCTGTGCCCTGGCTGCGTTAATT
TGCTTCGTCATTAGGTTGGCGAAGAACTGTATGTCCTGGCGCTACTCATGCACCAGATACACCAACTTTCTTC
TGGACACTAAGGGCAGACTCTATCGTTGGCGGTCGCCTGTCATCATAGAGAAAGGGGGTAAGGTAGAGGT
CGAAAGCCATCTGATCGACCTCAAAAGAGTTGTGCTTGATGGGTCCGCGGCAACCCCTTTAACCAGAGTTTC
AGCGGAACAATGGGGTCGTCCCTAG

>AF176348.2_Sublineage_5A(CANADA-PA8)

ATGTTGGGGAAATGCTTGACCGCGGGCTGGTGCTCGCAATTGCTTTCTTTGGGGTGTATCGTGCCGTTCTGT
TTTGCTGTGCTCGCCAACGCCAGCAACGACAGCAGCTCCCATGTACAGCTGATTTACAACTTGACGCTATGT
GAGCTGAATGGCACAGATTGGCTAGCTAACAAATTTGATTGGGCAGTGGAGAGTTTTGTCATCTTTCCCGTT
TTGACTCACATTGTCTCCTATGGTGCCCTCACTACCAGCCATTTCCTTGACACAGTCGCTTTAGTCACTGTGTC
TACCGCCGGGTTTGTTCACGGGCGGTATGTCCTAAGTAGCATCTACGCGGTCTGTGCCCTGGCTGCGTTGAC
TTGCTTCGTCATTAGGTTTGCAAAGAATTGCATGTCCTGGCGCTACGCGTGTACCAGATATACCAACTTTCTT
CTGGACACTAAGGGCAGACTCTATCGTTGGCGGTCGCCTGTCATCATAGAGAAAAGGGGCAAAGTTGAGG
TCGAAGGTCATCTGATCGACCTCAAAAGAGTTGTGCTTGATGGTTCCGTGGCAACCCCTATAACCAGAGTTT
CAGCGGAACAATGGGGTCGCCCTTAG

>AF066183.4_Sublineage_5A(RespPRRS_MLV)

ATGTTGGAGAAATGCTTGACCGCGGGCTGTTGCTCGCAATTGCTTTCTTTGTGGTGTATCGTGCCGTTCTGT
TTTGCTGTGCTCGCCAACGCCAGCAACGACAGCAGCTCCCATCTACAGCTGATTTACAACTTGACGCTATGT
GAGCTGAATGGCACAGATTGGCTAGCTAACAAATTTGATTGGGCAGTGGAGAGTTTTGTCATCTTTCCCGTT
TTGACTCACATTGTCTCCTATGGTGCCCTCACTACCAGCCATTTCCTTGACACAGTCGCTTTAGTCACTGTGTC
TACCGCCGGGTTTGTTCACGGGCGGTATGTCCTAAGTAGCATCTACGCGGTCTGTGCCCTGGCTGCGTTGAC
TTGCTTCGTCATTAGGTTTGCAAAGAATTGCATGTCCTGGCGCTACGCGTGTACCAGATATACCAACTTTCTT
CTGGACACTAAGGGCGGACTCTATCGTTGGCGGTCGCCTGTCATCATAGAGAAAAGGGGCAAAGTTGAGG
TCGAAGGTCATCTGATCGACCTCAAAAGAGTTGTGCTTGATGGTTCCGTGGCAACCCCTATAACCAGAGTTT
CAGCGGAACAATGGGGTCGTCCTTAG
