# Supplementary material for: Genetic variability, N-glycosylation, and recombination in sublineage 1A of Betaarterivirus americense from commercial pig farms in Lima, 2019
Source: Front Microbiol. 2026 May 18;17:1803991. doi: 10.3389/fmicb.2026.1803991 (PMC13224472; doi:10.3389/fmicb.2026.1803991)
Supplement: Supplementary Material 3 — Sequence data submitted to GenBank under BankIt identifiers 25–48. [file Supplementary_file_3.pdf]

Bankit/filename sequence ID identifier	Sequence
RC05088925.1_montana/PERU/2019	>RC05088925.1_montana/PERU/2019<br>ATGTTGGGGAAATGCTTGACCGCGGGCTGCTGCTCGCAATTGCTTTTTTTGTGGTGTATCGTGCCGTTCTGTTTTGTTGCGCTCGTCAACGCCAGCAA<br>CAGCAGCAGCTCCCATTTTCAGTTGATTTATAACCTGACGATATGCGAGCTGAATGGCACAGATTGGCTAAATAGAAGTTTTGATTGGGCGGTAGAG<br>ACCTTTGTTATCTTTCCTGTGTTGACTCATATTGTCTCCTATGGCGCCCTCACCACCAGCCATTTCCTTGACACAGTCGGCCTGATCACCGTGTCTGCCG<br>CCGGATATTACCACGGGCGGTATGTCTTGAGTAGCATTTATGCCGTCTGCGCCCTGGCTGCGTTAACTTGCTTTGTCATCAGGCTAACAAAAAATTGC<br>ATGTCCTGGCGTTACTCGTGCACCAGGTACACTAACTATCTTCTGGACACTAAGGGCAAACTCTATCGTTGGCGGTCTCCTGTCATCATAGAGAAAGG<br>GGGTAAAATTGAGGTCGAAGGTCACCTGATCGACCTCAAGAGAGTTGTGCTTGACGGTTCCGCGGCAACCCCTGTAACCAAAGTTTCAGCGGAACA<br>ATGGGGTCGTCCTTAG
RC05088926.1_montana/PERU/2019	>RC05088926.1_montana/PERU/2019<br>ATGTTGGGGAAATGCTTGACCGCGGGCTGCTGCTCGCAATTGCTTTTTTTGTGGTGTATCGTGCCGTTCTGTTTTGTTGCGCTCGTCAACGCCAGCAA<br>CAGCAGCAGCTCCCATTTTCAGTTGATTTATAACCTGACGATATGCGAGCTGAATGGCACAGATTGGCTAAATAGAAGTTTTGATTGGGCGGTAGAG<br>ACCTTTGTTATCTTTCCTGTGTTGACTCATATTGTCTCCTATGGCGCCCTCACCACCAGCCATTTCCTTGACACAGTCGGCCTGATCACCGTGTCTGCCG<br>CCGGATATTACCACGGGCGGTATGTCTTGAGTAGCATTTATGCCGTCTGCGCCCTGGCTGCGCTAACTTGCTTTGTCATCAGGCTAACAAAAAATTGC<br>ATGTCCTGGCGTTACTCGTGCACCAGGTACACTAACTATCTTCTGGACACTAAGGGCAAACTCTATCGTTGGCGGTCTCCTGTCATCATAGAGAAAGG<br>GGGTAAAATTGAGGTCGAAGGTCACCTGATCGACCTCAAGAGAGTTGTGCTTGACGGTTCCGCGGCAACCCCTGTAACCAAAGTTTCAGCGGAACA<br>ATGGGGTCGTCCTTAG
RC05088927.1_montana/PERU/2019	>RC05088927.1_montana/PERU/2019<br>ATGTTGGGGAAATGCTTGACCGCGGGCTGCTGCTCGCAATTGCTTTTTTTGTGGTGTATCGTGCCGTTCTGTTTTGTTGCGCTCGTCAACGCCAGCAA<br>CAGCAGCAGCTCCCATTTTCAGTTGATTTATAACCTGACGATATGCGAGCTGAATGGCACAGATTGGCTAAATAGAAGTTTTGATTGGGCGGTAGAG<br>ACCTTTGTTATCTTTCCTGTGTTGACTCATATTGTCTCCTATGGCGCCCTCACCACCAGCCATTTCCTTGACACAGTCGGCCTGATCACCGTGTCTGCCG<br>CCGGATATTACCACGGGCGGTATGTCTTGAGTAGCATTTATGCCGTCTGCGCCCTGGCTGCGTTAACTTGCTTTGTCATCAGGCTAACAAAAAATTGC<br>ATGTCCTGGCGTTACTCGTGCACCAGGTACACTAACTATCTTCTGGACACTAAGGGCAAACTCTATCGTTGGCGGTCTCCTGTCATCATAGAGAAAGG<br>GGGTAAAATTGAGGTCGAAGGTCACCTGATCGACCTCAAGAGAGTTGTGCTTGACGGTTCCGCGGCAACCCCTGTAACCAAAGTTTCAGCGGAACA<br>ATGGGGTCGTCCTTAG

RC05088928.1_montana/PERU/2019	>RC05088928.1_montana/PERU/2019<br>ATGTTGGGGAAATGCTTGACCGCGGGCTGCTGCTCGCAATTGCTTTTTTTGTGGTGTATCGTGCCGTTCTGTTTTGTTGCGCTCGTCAACGCCAGCAA<br>CAGCAGCAGCTCCCATTTTCAGTTGATTTATAACCTGACGATATGCGAGCTGAATGGCACAGATTGGCTAAATAGAAGTTTTGATTGGGCGGTAGAG<br>ACCTTTGTTATCTTTCCTGTGTTGACTCATATTGTCTCCTATGGCGCCCTCACCACCAGCCATTTCCTTGACACAGTCGGCCTGATCACCGTGTCTGCCG<br>CCGGATATTACCACGGGCGGTATGTCTTGAGTAGCATTTATGCCGTCTGCGCCCTGGCTGCGTTAACTTGCTTTGTCATCAGGCTAACAAAAAATTGC<br>ATGTCCTGGCGTTACTCGTGCACCAGGTACACTAACTATCTTCTGGACACTAAGGGCAAACTCTATCGTTGGCGGTCTCCTGTCATCATAGAGAAAGG<br>GGGTAAAATTGAGGTCGAAGGTCACCTGATCGACCTCAAGAGAGTTGTGCTTGACGGTTCCGCGGCAACCCCTGTAACCAAAGTTTCAGCGGAACA<br>ATGGGGTCGTCCTTAG
RC05088929.1_montana/PERU/2019	>RC05088929.1_montana/PERU/2019<br>ATGTTGGGGAAATGCTTGACCGCGGGCTGCTGCTCGCAATTGCTTTTTTTGTGGTGTATCGTGCCGTTCTGTTTTGTTGCGCTCGTCAACGCCAGCAA<br>CAGCAGCAGCTCCCATTTTCAGTTGATTTATAACCTGACGATATGCGAGCTGAATGGCACAGATTGGCTAAATAGAAATTTTGATTGGGCGGTAGAG<br>ACCTTTGTTATCTTTCCTGTGTTGACTCATATTGTCTCCTATGGCGCCCTCACCACCAGCCATTTCCTTGACACAGTCGGCCTGATCACCGTGTCTGCCG<br>CCGGATATTACCACGGGCGGTATGTCTTGAGTAGCATTTATGCCGTCTGCGCCCTGGCTGCATTAATTTGCTTTGTCATCAGGCTAACAAAAAATTGC<br>ATGTCCTGGCGTTACTCGTGCACCAGGTACACTAACTATCTTCTGGACACTAAGGGCAAACTCTATCGTTGGCGGTCTCCTGTCATCATAGAGAAAGG<br>GGGTAAAATTGAGGTCGAAGGTCACCTGATCGACCTCAAGAGAGTTGTGCTTGACGGTTCCGCGGCAACCCCTGTAACCAAAGTTTCAGCGGAACA<br>ATGGGGTCGTCCTTAG
RC05088930.1_montana/PERU/2019	>RC05088930.1_montana/PERU/2019<br>ATGTTGGGGAAATGCTTGACCGCGGGCTGCTGCTCGCAATTGCCTTTTTTGTGGTGTATCGTGCCGTTCTGTTTTGTTGTGCTCGTCAACGCCAACAA<br>CAGCAACAGCTCCCATTTACAGTTGATTTATAACCTGACGATATGTGAGCTGAATGGCACAGATTGGCTAAATGAAAGTTTTGATTGGGCGGTGGAG<br>ACCTTTGTTATCTTTCCTGTGTTGACTCATATTGTCTCCTACGGCGCCCTCACCACCAGCCATTTCCTTGACACAGTCGGCCTGATCACCGTGTCTGCCG<br>CCGGATACTACCACGGACGGTATGTCTTAAGTAGCATTTACGCCGTCTGCGCCATGGCTGCGTTAACTTGCTTCGTCATCAGGCTAACAAAAAATTGT<br>ATGTCCTGGCGTTACTCATGTACCAGGTACACTAATTTTCTTCTGGACACCAAGGGCAAACTCTATCGTTGGCGGTCTCCTGTCATCATAGAGAAAGG<br>GGGTAAAGTTGAGGTCGAAGGTCACCTGATCGACCTCAACAGAGTTGTGCTTGACGGTTCCGCGGCAACCCCTGTAACCAAAGTTTCAGCGGAACA<br>ATGGGGTCGTCCTTAG
RC05088931.1_montana/PERU/2019	>RC05088931.1_montana/PERU/2019<br>ATGTTGGGGAAATGCTTGACCGCGGGCTGCTGCTCGCAATTGCCTTTTTTGTGGTGTATCGTGCCGTTCTGTTTTGTTGTGCTCGTCAACGCCAACAA<br>CAGCAACAGCTCCCATTTACAGTTGATTTATAACCTGACGATATGTGAGCTGAATGGCACAGATTGGCTAAATAAAAGTTTTGATTGGGCGGTGGAG<br>ACCTTTGTTATCTTTCCTGTGTTGACTCATATTGTCTCCTACGGCGCCCTCACCACCAGCCATTTCCTTGACACAGTCGGCCTGATCACCGTGTCTGCCG<br>CCGGATACTACCACGGACGGTATGTCTTAAGTAGCATTTACGCCGTCTGCGCCATGGCTGCGTTAACTTGCTTCGTCATCAGGCTAACAAAAAATTGT<br>ATGTCCTGGCGTTACTCATGTACCAGGTACACTAATTTTCTTCTGGACACCAAGGGCAAACTCTATCGTTGGCGGTCTCCTGTCATCATAGAGAAAGG<br>GGGTAAAGTTGAGGTCGAAGGTCACCTGATCGACCTCAAGAGAGTTGTGCTTGACGGTTCCGCGGCAACCCCTGTAACCAAAGTTTCAGCGGAACA<br>ATGGGGTCGTCCTTAG

RC05088932.1_montana/PERU/2019	>RC05088932.1_montana/PERU/2019<br>ATGTTGGGGAAATGCTTGACCGCGGGCTGCTGCTCGCAATTGCCTTTTTTGTGGTGTATCGTGCCGTTCTGTTTTGTTGTGCTCGTCAACGCCAACAA<br>CAGCAACAGCTCCCATTTACAGTTGATTTATAACCTGACGATATGTGAGCTGAATGGCACAGATTGGCTAAATAAAAGTTTTGATTGGGCGGTGGAG<br>ACCTTTGTTATCTTTCCTGTGTTGACTCATATTGTCTCCTACGGCGCCCTCACCACCAGCCATTTCCTTGACACAGTCGGCCTGATCACCGTGTCTGCCG<br>CCGGATACTACCACGGACGGTATGTCTTAAGTAGCATTTACGCCGTCTGCGCCATGGCTGCGTTAACTTGCTTCGTCATCAGGCTAACAAAAAATTGT<br>ATGTCCTGGCGTTACTCATGCACCAGATACACTAATTTTCTTCTGGACACCAAGGGCAAACTCTATCGTTGGCGGTCTCCTGTCATCATAGAGAAAGG<br>GGGTAAAGTTGAGGTCGAAGGTCACCTGATCGACCTCAAGAGAGTTGTGCTTGACGGTTCCGCGGCAACCCCTGTAACCAAAGTTTCAGCGGAACA<br>ATGGGGTCGTCCTTAG
RC05088933.1_montana/PERU/2019	>RC05088933.1_montana/PERU/2019<br>ATGTTGGGGAAATGCTTGACCGCGGGCTGCTGCTCGCAATTGCCTTTTTTGTGGTGTATCGTGCCGTTCTGTTTTGTTGTGCTCGTCAACGCCAACAA<br>CAGCAACAGCTCCCATTTACAGTTGATTTATAACCTGACGATATGTGAGCTGAATGGCACAGATTGGCTAAATAAAAGTTTTGATTGGGCGGTGGAG<br>ACCTTTGTTATCTTTCCTGTGTTGACTCATATTGTCTCCTACGGCGCCCTCACCACCAGCCATTTCCTTGACACAGTCGGCCTGATCACCGTGTCTGCCG<br>CCGGATACTACCACGGACGGTATGTCTTAAGTAGCATTTACGCCGTCTGCGCCATGGCTGCGTTAACTTGCTTCGTCATCAGGCTAACAAAAAATTGT<br>ATGTCCTGGCGTTACTCATGTACCAGGTACACTAATTTTCTTCTGGACACCAAGGGCAAACTCTATCGTTGGCGGTCTCCTGTCATCATAGAGAAAGG<br>GGGTAAAGTTGAGGTCGAAGGTCACCTGATCGACCTCAAGAGAGTTGTGCTTGACGGTTCCGCGGCAACCCCTGTAACCAAAGTTTCAGCGGAACA<br>ATGGGGTCGTCCTTAG
RC05088934.1_montana/PERU/2019	>RC05088934.1_montana/PERU/2019<br>ATGTTGGGGAAATGCTTGACCGCGGGCTGCTGCTCGCAATTGCTTTTTTTGTGGTGTATCGTGCCGTTCTGTTTTGTTGCGCTCGTCAACGCCAGCAA<br>CAGCAGCAGCTCCCATTTTCAGTTGATTTATAACCTGACGATATGCGAGCTGAATGGCACAGATTGGCTAAATAGAAGTTTTGATTGGGCGGTAGAG<br>ACCTTTGTTATCTTTCCTGTGTTGACTCATATTGTCTCCTATGGCGCCCTCACCACCAGCCATTTCCTTGACACAGTCGGCCTGATCACCGTGTCTGCCG<br>CCGGATATTACCACGGGCGGTATGTCTTGAGTAGCATTTATGCCGTCTGCGCCCTGGCTGCGTTAACTTGCTTTGTCATCAGGCTAACAAAAAATTGC<br>ATGTCCTGGCGTTACTCGTGCACCAGGTACACTAACTATCTTCTGGACACTAAGGGCAAACTCTATCGTTGGCGGTCTCCTGTCATCATAGAGAAAGG<br>GGGTAAAATTGAGGTCGAAGGTCACCTGATCGACCTCAAGAGAGTTGTGCTTGACGGTTCCGCGGCAACCCCTGTAACCAAAGTTTCAGCGGAACA<br>ATGGGGTCGTCCTTAG

RC05088935.1_montana/PERU/2019	>RC05088935.1_montana/PERU/2019<br>ATGTTGGGGAAATGCTTGACCGCGGGCTGCTGCTCGCAATTGCTTTTTTTGTGGTGTATCGTGCCGTTCTGTTTTGTTGCGCTCGTCAACGCCAGCAA<br>CAGCAGCAGCTCCCATTTTCAGTTGATTTATAACCTGACGATATGCGAGCTGAATGGCACAGATTGGCTAAATAGAAGTTTTGATTGGGCGGTAGAG<br>ACCTTTGTTATCTTTCCTGTGTTGACTCATATTGTCTCCTATGGCGCCCTCACCACCAGCCATTTCCTTGACACAGTCGGCCTGATCACCGTGTCTGCCG<br>CCGGATATTACCACGGGCGGTATGTCTTGAGTAGCATTTATGCCGTCTGCGCCCTGGCTGCGTTAACTTGCTTTGTCATCAGGCTAACAAAAAATTGC<br>ATGTCCTGGCGTTACTCGTGCACCAGGTACACTAACTATCTTCTGGACACTAAGGGCAAACTCTATCGTTGGCGGTCTCCTGTCATCATAGAGAAAGG<br>GGGTAAAATTGAGGTCGAAGGTCACCTGATCGACCTCAAGAGAGTTGTGCTTGACGGTTCCGCGGCAACCCCTGTAACCAAAGTTTCAGCGGAACA<br>ATGGGGTCGTCCTTAG
RC05088936.1_montana/PERU/2019	>RC05088936.1_montana/PERU/2019<br>ATGTTGGGGAAATGCTTGACCGCGGGCTGCTGCGCGCGGGGGCTTTTTTGGTGGTGGATCGATAAGTTCTGTTTGGTTTCGCTCGTCAATGCCGAG<br>AACAGCAGCAGCTCCCATTTAAGATAAATCCATATTCGGGCGGTATGCGGTCTGAATGGCACAAATTGGCTAAATAGAAGGTTTGATTGGGCGGTG<br>TAGACCTTTGTTGTCTTTCCTGTGTTGACTCATATTGTCTCCTATGGCGCCCTCACCACCAGCCATTTCCTTGACACAGTCGGTTTGATGAAGGTGTCT<br>GCCGCCGGATATTACCACGGGCAGCGTGTCAAAAGTAGCATTTACGCCGTGTGCGCCCTGGCTGCGATGGGCCGATTTGTCATCAGACAACGAAAA<br>AATTGTACCTCCGGGCGGGACTCACGCACCAGATACGCTAACTATCTTCTGGACACTAAGGGCAAACTATATCGTTGGCGGTCTCCTGTCATCATAG<br>AGAAAGGGGGTAAAATTGAGGTTGGAGGTCACCTGATCGACCTCAAGAGAGTTGTGCTTGACGGTTCCGCGGCGACTCCTGTAACCAAAGTTTCAG<br>CGGAACAATGGGGTCGTCCTTAG
RC05088937.1_montana/PERU/2019	>RC05088937.1_montana/PERU/2019<br>ATGTTGGGGAAATGCTTGACCGCGGGCTGCTGCGCGCGGGGGCTTTTTTGGTGGTGGATCGATAAGTTCTGTTTGGTTTCGCTCGTCAATGCCGAG<br>AACAGCAGCAGCTCCCATTTAAGATAAATCCATATTCGGGCGGTATGCGGTCTGAATGGCACAAATTGGCTAAATAGAAGGTTTGATTGGGCGGTG<br>TAGACCTTTGTTGTCTTTCCTGTGTTGACTCATATTGTCTCCTATGGCGCCCTCACCACCAGCCATTTCCTTGACACAGTCGGTTTGATTAAGGTGTCTG<br>CCGCCGGATATTACCACGGACGGCATGTCAAAAGTAGCATTTACGCCGTGTGCGCCCTGGCTGCGATGGGCCGATTTGTCATCAGACAACGAAAAA<br>ATTGCACCTCCGGGCGGGACTCACGCACCAGATACGCTAACTATCTTCTGGACACCAAGGGCAAACTATATCGTTGGCGGTCTCCTGTCATCATAGA<br>GAAAGGGGGCAAAATTGAGGTTGTCTTTCACCTGATCGACCTCAAGAGAGTTGTGCTTGACGGTTCCGCGGCGACTCCTGTACCCTAAGTTTCAGCG<br>GAACAATGGGGTCGTCCTTAG

RC05088938.1_montana/PERU/2019	>RC05088938.1_montana/PERU/2019<br>ATGTTGGGGAAATGCTTGACCGCGGGCTGCTGCGCGCGGGGGCTTTTTTGGTGGTGGATCGATAAGTTCTGTTTGGTTTCGCTCGTCAATGCCGAG<br>AACAGCAGCAGCTCCCATTTAAGATAAATCCATATTCGGGCGGTATGCGGTCTGAATGGCACAAATTGGCTAAATAGAAGGTTTGATTGGGCGGTG<br>TAGACCTTTGTTGTCTTTCCTGTGTTGACTCATATTGTCTCCTATGGCGCCCTCACCACCAGCCATTTCCTTGACACAGTCGGTTTGATGAAGGTGTCT<br>GCCGCCGGATATTACCACGGGCGGCATGTCAAAAGTAGCATTTACGCCGTGTGCGCCCTGGCTGCGATGGGCCGATTTGTCATCAGACTAACAAAA<br>AATTGTACCTCCGGGCGGGACTCACGCACCAGATACGCTAACTATCTTCTGGACACTAAGGGCAAACTATATCGTTGGCGGTCTCCTGTCATCATAG<br>AGAAAGGGGGTAAAATTGAGGTCGAAGGTCACCTGATCGACCTCAAGAGAGTTGTGCTTGACGGTTCCGCGGCGACTCCTGTAACCAAAGTTTCAG<br>CGGAACAATGGGGTCGTCCTTAG
RC05088939.1_montana/PERU/2019	>RC05088939.1_montana/PERU/2019<br>ATGTTGGGGAAATGCTTGACCGCGGGCTGCTGCGCGCGGGGGCTTTTTTGGTGGTGGATCGATAAGTTCTGTTTGGTTTCGCTCGTCAATGCCGAG<br>AACAGCAGCAGCTCCCATTTAAGATAAATCCATATTCGGGCGGTATGCGGTCTGAATGGCACAAATTGGCTAAATAGAAGGTTTGATTGGGCGGTG<br>TAGACCTTTGTTGTCTTTCCTGTGTTGACTCATATTGTCTCCTATGGCGCCCTCACCACCAGCCATTTCCTTGACACAGTCGGTTTGATTAAGGTGTCTG<br>CCGCCGGATATTACCACGGGCGGCATGTCAAAAGTAGCATTTACGCCGTGTGCGCCCTGGCTGCGATGGGCCGATTTGTCATCAGACTAACAAAAA<br>ATTGCACCTCCGGGCGGGACTCACGCACCAGATACGCTAACTATCTTCTGGACACCAAGGGCAAACTATATCGTTGGCGGTCTCCTGTCATCATAGA<br>GAAAGGGGGTAAAATTGAGGTCGAAGGTCACCTGATCGACCTCAAGAGAGTTGTGCTTGACGGTTCCGCGGCGACTCCTGTACCCTAAGTTTCAGC<br>GGAACAATGGGGTCGTCCTTAG
RC05088940.1_montana/PERU/2019	>RC05088940.1_montana/PERU/2019<br>ATGTTGGGGAAATGCTTGACCGCGGGCTGCTGCTCGCAATTGCCTTTTTTGTGGTGTATCGTGCCGTTCTGTTTTGTTGCGCTCGTCAACGCCAGCAA<br>CAACAGCAGCTCCCATTTACAGTTGATTTATAACCTGACGATATGTGAGCTGAACGGCACAGATTGGCTAAATAAAAGTTTTGATTGGGCGGTGGAG<br>ACCTTTGTTATCTTTCCTGTGTTGACTCATATTGTCTCCTATGGCGCCCTCACCACCAGCCATTTCCTTGACACAGTCGGCCTGATCACCGTGTCTGCCG<br>CCGGATATTACCACGGACGGTATGTTTTGAGTAGCATTTACGCCGTCTGCGCCCTGGCTGCGTTAACTTGCTTCATCATCAGGCTAACAAAAAATTGT<br>ATGTCCTGGCGTTACTCATGCACCAGGTACACTAATTTTCTTCTGGACACCAAGGGCAAACTCTATCGTTGGCGGTCTCCTGTCATCATAGAGAAAGG<br>GGGTAAAGTTGAGGTCGAAGGTCACCTGATCGACCTCAAGAGAGTTGTACTTGACGGTTCCGCGGCTACCCCTGTAACCAAAGTTTCAGCGGAACA<br>ATGGGGTCGTCCTTAG

RC05088941.1_montana/PERU/2019	>RC05088941.1_montana/PERU/2019<br>ATGTTGGGGAAATGCTTGACCGCGGGCTGCTGCTCGCAATTGCCTTTTTTGTGGTGTATCGTGCCGTTCTGTTTTGTTGCGCTCGTCAACGCCAGCAA<br>CAACAGCAGCTCCCATTTACTGTTGATTTATAACCTGACGATATGTGAGCTGAACGGCACAGATTGGCTAAATAAAAGTTTTGATTGGGCGGTGGAG<br>ACCTTTGTTATCTTTCCTGTGTTGACTCATATTGTCTCCTATGGCGCCCTCACCACCAGCCATTTCCTTGACACAGTCGGCCTGATCACCGTGTCTGCCG<br>CCGGATATTACCACGGACGGTATGTTTTGAGTAGCATTTACGCCGTCTGCGCCCTGGCTGCGTTAACTTGCTTCATCATCAGGCTAACAAAAAATTGT<br>ATGTCCTGGCGTTACTCATGCACCAGGTACACTAATTTTCTTCTGGACACCAAGGGCAAACTCTATCGTTGGCGGTCTCCTGTCATCATAGAGAAAGG<br>GGGTAAAGTTGACGTCGAAGGTCACCTGATCGACCTCAAGAGAGTTGTACTTGACGGTTCCGCGGCTACCCCTGTAACCAAAGTTTCAGCGGAACA<br>ATGGGGTCGTCCTTAG
RC05088942.1_montana/PERU/2019	>RC05088942.1_montana/PERU/2019<br>ATGTTGGGGAAATGCTTGACCGCGGGCTGCTGCTCGCAATTGCCTTTTTTGTGGTGTATCGTGCCATTCTGTTTTGTTGCGCTCGTCAACACCCGCAA<br>CACCAGCATCTCCCTTTTACTTTTGATTTATAACCTGACGATATGTGAGCTGAATGGCACAGATTGGCTAAATAAAAGCTTTGATTGGGCGGTGGAGA<br>CCTTTGTTATCTTTCCTGTGTTGACTCATATTGTCTCCTATGGCGCCCTCACCACCAGCCATTTCCTTGACACAGTCGGCCTGATCACCGTGTCTGCCGC<br>CGGATATTACCACGGACGGTATGTTTTAAGTAGCATTTACGCCGTCTGCGCCCTGGCTGCGTTAACTTGCTTCGTCATCTCGCTATCTAAAAATTGTAT<br>GTCCTGGCGTTATTCTTGCACCAAGTACACTACTTTTCTTCTGGACACCAAGTGCAATCTCTCTCGCGGAAGTTCTCCTGTGATGATACACCGCTGGGG<br>TTAAAACGAAGTCTCATGTTTCCTGTCCCTCTTCAAAAGAGTTGTGCTTGACGGTTCCGCGGCTACCCCTGTAACCAAAGTTTCAGCGGAACAATGGG<br>GTCGTCCTTAG
RC05088943.1_montana/PERU/2019	>RC05088943.1_montana/PERU/2019<br>ATGTTGGGGAAATGCTTGACCGCGGGCTGCTGCTCGCAATTGCCTTTTTTGTGGTGTATCGTGCCGTTCTGTTTTGTTGCGCTCGTCAACGCCGGCAA<br>CAGCATCAGCTCCCATTTACAGGTGATTTATAACCTGACGATATGTGAGCTGAATGGCACAGATTGGCTAAATAAAAGCTTTGATTGGGCGGTGGAG<br>ACCTTTGTTATCTTTCCTGTGTTGACTCATATTGTCTCCTATGGCGCCCTCACCACCAGCCATTTCCTTGACACAGTCGGCCTGATCACCGTGTCTGCCG<br>CCGGATATTACCACGGACGGTATGTTTTGAGTAGCATTTACGCCGTCTGCGCCCTGGCTGCATTAACTTGCTTCGTCATCAGGCTAACAAAAAATTGT<br>ATGTCCTGGCGTTATTCATGCACCAGGTACACTAATTTTCTTCTGGACACCAAGGGCAAACTCTATCCTTGGCGGTCTCCTGTCATCATAAAGAAAGG<br>GGGGAAAATAGAGGTAGAAGGTCACATGATCGACCTCAAGAGAGTTGTACTTGACGGTTCCGCGGCTACCCCTGTAACCAAAGTTTCAGCGGAACA<br>ATGGGGTCGTCCTTAG

RC05088944.1_montana/PERU/2019	>RC05088944.1_montana/PERU/2019<br>ATGTTGGGGAAATGCTTGACCGCGGGCTGCTGCTCGCAATTGCCTTTTTTGTGGTGTATCGTGCCGTTCTGTTTTGTTGCGCTCGTCAACGCCGGCAA<br>CAGCATCAGCTCCCATTTACAGGTGATTTATAACCTGACGATATGTGAGCTGAATGGCACAGATTGGCTAAATAAAAGCTTTGATTGGGCGGTGGAG<br>ACCTTTGTTATCTTTCCTGTGGTGACTCATATTGTCTCCTATGGCGCCTTCACCACCAGCCATTTCCTTGACACAGTCGGCCTGATCACCGTGTCTGCCG<br>CCGGATATTACCCCGGACGGTATGTTTTGAGTAGCATTTACGCCGTTTGCGCCCTGGATGCGTTAACTTGCTTCGTCATCAGGCTAACAAAAAATTGT<br>ATGTCCTGGCGTTATTCATGCACCAGGTACAGTAATTTTCTTCTGGACACCAAGGGCAAACTCTATCGTTGGCGGTCTCCTGTCATCATAGAGAAAGG<br>GGGTAAAATAGAGGTAGAAGGTCACATGATCGACCTCAAGAGAGTTGTACTTGACGGTTCCGCGGCTACCCCTGTAACCAAAGTTTCAGCGGAACA<br>ATGGGGTCGTCCTTAG
RC05088945.1_montana/PERU/2019	>RC05088945.1_montana/PERU/2019<br>GTGTTGGGGAAATGCTTGACCGCGGGCTGCTGCTCGCAATTGCCTTTTTTGTGGTGTATCGTGCCGTTCTGTTTTGTTGTGCTCGTCAACGCCAACAA<br>CAGCAGCAGCTCCCATTTACAGTATGTTTATAACCTGACGATATGTGAGCTGAATGGCACAGATTGGCTAAACGTTCATTTTGATTGGGCGGTGGAG<br>ACCTTTGTCATCTTTCCTGTGTTGACTCACATTGTCTCCTTTGGTGCCCTTACCACCAGCCATTTCCTTGACACATTCGGCCTGATCACCGTGTCTACCG<br>CCGGATATTACCACGGGCGGTATGTCTTGAGTAGCATTTACGCCGTCTGCGCCCTAGCCGCGTTAATTTGCTTCATCATCAAGCTAACAAAAAATTGT<br>ATGTCCTGGCGTTACTCATGCACCAGGTACACTAATTTTCATCTGGACACCAAGGGCAAACTCTATCGTTGGCGGTCTCCTGTCATCATAGAGAAAGG<br>GGGTAAAGTTGAGGTCCAAGGTCACCTGATAGACCTCAAAAGAGTTGTGCTTGACGGTTCCGCGGCTACCCCTGTAACCAAAGTTTCAGCGGAACA<br>ATGGGGTCGTCCTTAG
RC05088946.1_montana/PERU/2019	>RC05088946.1_montana/PERU/2019<br>GTGTTGGGGAAATGCTTGACCGCGGGCTGCTGCTCGCAATTGCCTTTTTTGTGGTGTATCGTGCCGTTCTGTTTTGTTGTGCTCGTCAACGCCAACAA<br>CAGCAGCAGCTCCCATTTACAGTTGATTTATAACCTGACGATATGTGAGCTGAATGGCACAGATTGGCTAAACGAACATTTTGATTGGGCGGTGGAG<br>ACCTTTGTCATCTTTCCTGTGTTGACTCACATTGTCTCCTATGGTGCCCTCACCACCAGCCATTTCCTTGACACAGTCGGCCTGATCACCGTGTCTACCG<br>CCGGATATTACCACGGGCGGTATGTCTTGAGTAGCATTTACGCCGTCTGCGCCCTAGCTGCGTTAACTTGCTTCATCATCAGGCTAACGAAAAACTGT<br>ATGTCCTGGCGTTACTCATGCACCAGGTACACTAATTTTCTTCTGGACACCAAGGGCAAACTCTATCGTTGGCGGTCTCCTGTCATCATAGAGAAAGG<br>GGGTAAAGTTGAGGTCGAAGGTCACCTGATCGACCTCAAGAGAGTTGTACTTGACGGTTCCGCGGCTACCCCTGTAACCAAAGTTTCAGCGGAACA<br>ATGGGGTCGTCCTTAG

RC05088947.1_montana/PERU/2019	>RC05088947.1_montana/PERU/2019<br>GTGTTGGGGAAATGCTTGACCGCGGGCTGCTGCTCGCAATTGCCTTTTTTGTGGTGTATCGTGCCGTTCTGTTTTGTTGTGCTCGTCAACGCCAACAA<br>CAGCAGCAGCTCCCATTTGCAGTTGATTTATAACCTGACGATATGTGAGCTGAATGGCACAGATTGGCTAAACGAACATTTTGATTGGGCGGTGGAG<br>ACCTTTGTCATCTTTCCTGTGTTGACTCACATTGTCTCCTATGGTGCCCTCACCACCAGCCATTTCCTTGACACAGTCGGCCTGATCACCGTGTCTACCG<br>CCGGATATTACCACGGGCGGTATGTCTTGAGTAGCATTTACGCCGTCTGCGCCCTAGCTGCGTTAACTTGCTTCATCATCAGGCTAACGAAAAACTGT<br>ATGTCCTGGCGTTACTCATGCACCAGGTACACTAATTTTCTTCTGGACACCAAGGGCAAACTCTATCGTTGGCGGTCTCCTGTCATCATAGAGAAAGG<br>GGGTAAAGTTGAGGTCGAAGGTCACCTTATCGACCTCAAGAGAGTTGTACTTGACGGTTCCGCGGCTACCCCTGTAACCAAAGTTTCAGCGGAACA<br>ATGGGGTCGTCCTTAG
RC05088948.1_montana/PERU/2019	>RC05088948.1_montana/PERU/2019<br>GTGTTGGGGAAATGCTTGACCGCGGGCTGCTGCTCGCAATTGCCTTTTTTGTGGTGTATCGTGCCGTTCTGTCTTGTTGTGCTCGTCAACGCCAACAA<br>CAGCAGCAGCTCCCATTTACAGTTGATTTATAACCTGACGATATGTGAGCTGAATGGCACAGATTGGCTAAACAAAAGTTTTGATTGGGCGGTGGAG<br>ACCTTTGTCATCTTTCCTGTGTTGACTCATATTGTCTCCTATGGCGCCCTTACCACCAGTCATTTCCTTGACACAGTCGGCCTGATCACCGTGTCTGCCG<br>CCGGATATTACCACGGGCGGTATGTCTTGAGTAGCATTTACGCCGTCTGCGCCTTAGCCGCGTTAATTTGCTTCATCATCAAGCTAACAAAAAATTGT<br>ATGTCCTGGCGTTACTCATGCACCAGGTACACTAATTTTCTTCTGGACACCAAGGGCAAACTCTATCGTTGGCGGTCTCCTGTCATCATAGAGAAAGG<br>GGGTAAAGTTGAGGTCCAAGGTCACCTGATAGACCTCAAAAGAGTTGTGCTTGACGGTTCCGCGGCTACCCCTGTAACCAAAGTTTCAGCGGAACA<br>ATGGGGTCGTCCTTAG
