# Supplementary material for: Genetic variability, N-glycosylation, and recombination in sublineage 1A of Betaarterivirus americense from commercial pig farms in Lima, 2019
Source: Front Microbiol. 2026 May 18;17:1803991. doi: 10.3389/fmicb.2026.1803991 (PMC13224472; doi:10.3389/fmicb.2026.1803991)
Supplement: Supplementary Material 4 — In-house conventional RT-PCR assay targeting the ORF5 gene. [file Image_1.pdf]

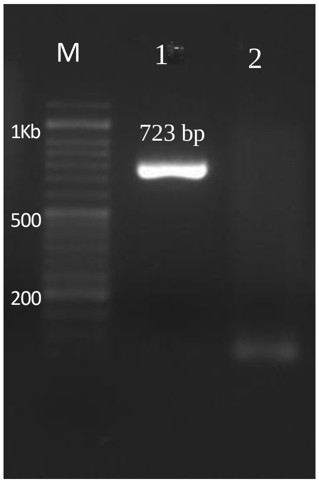

Supplementary Material 4. Conventional RT-PCR assay targeting the ORF5 gene developed in-house. Lane 1 (ORF5-SKA): cDNA from a PRRS-positive field sample; Lane 2 (negative control): nuclease-free water; Lane M (marker): Opti-DNA ABM® 100 bp DNA ladder (50 bp–1.5 kb).
