# Supplementary material for: Genetic variability, N-glycosylation, and recombination in sublineage 1A of Betaarterivirus americense from commercial pig farms in Lima, 2019
Source: Front Microbiol. 2026 May 18;17:1803991. doi: 10.3389/fmicb.2026.1803991 (PMC13224472; doi:10.3389/fmicb.2026.1803991)
Supplement: Supplementary Material 6 — Genomic recombination analysis of strain RC05088942.1_montana/PERU/2019-R of porcine reproductive and respiratory syndrome virus (PRRSV) from commercial farms in Lima, 2019. [file Image_3.pdf]

A

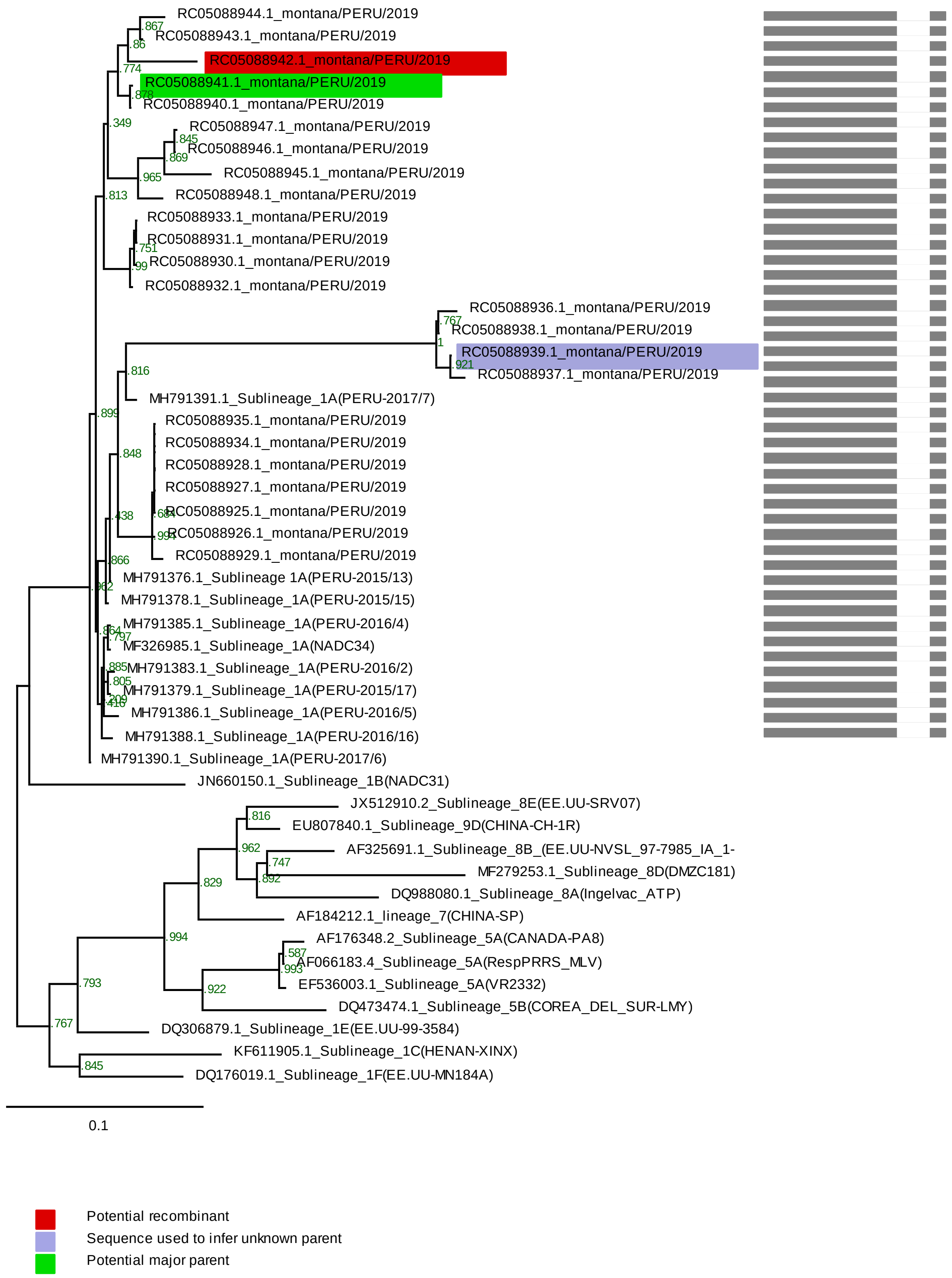

Supplementary Material 6a. Maximum-likelihood phylogenetic tree of the major parental sequence.

B

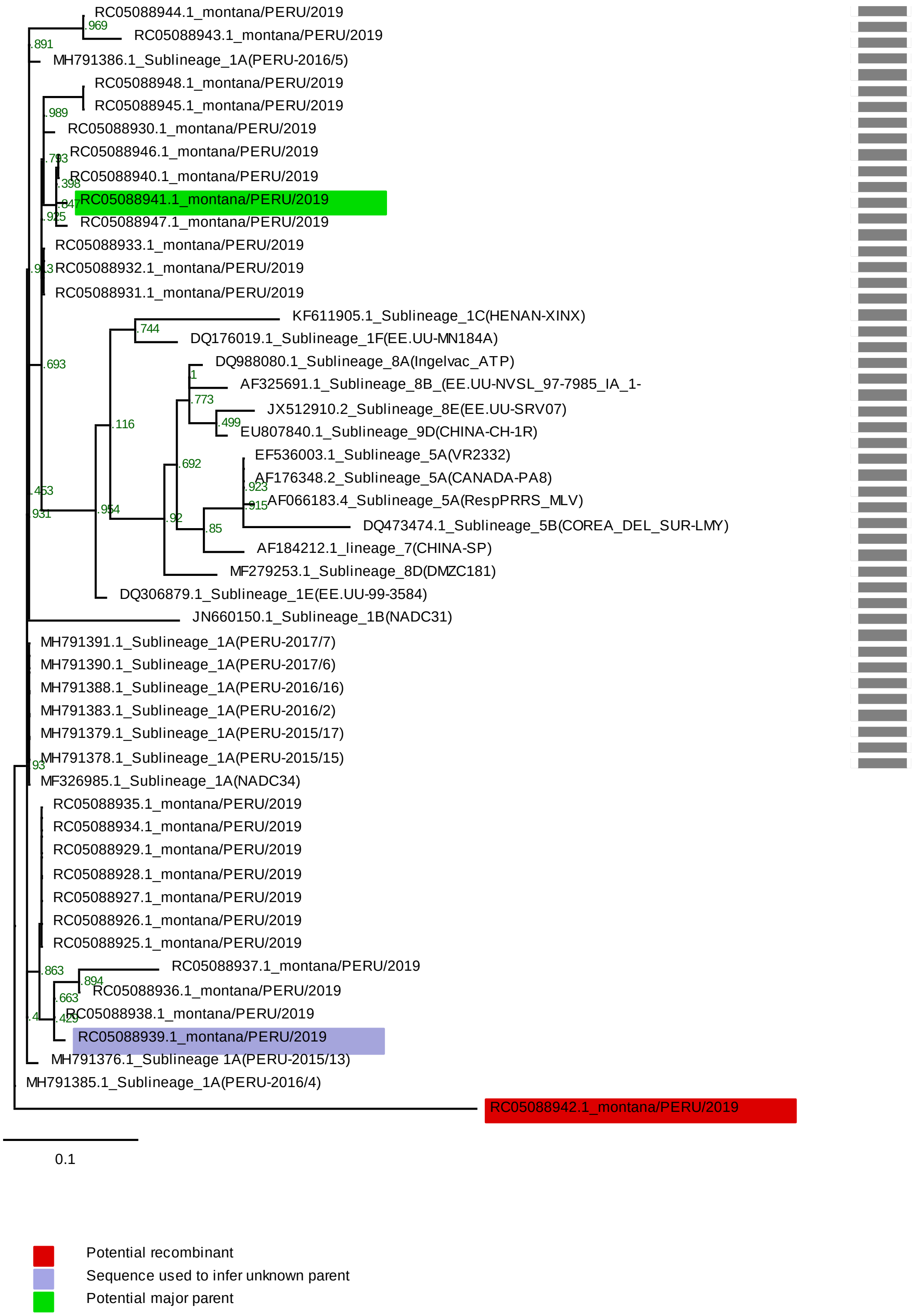

Supplementary Material 6b. Maximum-likelihood phylogenetic tree of the minor parental sequence.

Supplementary Material 6. Genomic recombination analysis of the RC05088942.1_montana/PERU/2019-R strain of porcine reproductive and respiratory syndrome virus (PRRSV) from commercial farms in Lima, 2019. (A) Maximum-likelihood phylogenetic tree of the region derived from the major parental strain RC05088941.1_montana/PERU/2019 (nt 1–439 and 550–603). (B) Maximum-likelihood phylogenetic tree of the region derived from the minor parental strain RC05088939.1_montana/PERU/2019 (nt 440–549). Parental phylogenies were inferred using RDP4.101 (http://web.cbio.uct.ac.za/~darren/rdp.html). The recombinant strain (RC05088942.1_montana/PERU/2019-R) is indicated in red; the major parental strain (RC05088941.1_montana/PERU/2019) in green; and the best candidate for the minor parental strain (RC05088939.1_montana/PERU/2019) in translucent blue. Scale bars represent nucleotide substitutions per site.
