# Supplementary material for: Genetic variability, N-glycosylation, and recombination in sublineage 1A of Betaarterivirus americense from commercial pig farms in Lima, 2019
Source: Front Microbiol. 2026 May 18;17:1803991. doi: 10.3389/fmicb.2026.1803991 (PMC13224472; doi:10.3389/fmicb.2026.1803991)
Supplement: Supplementary Material 5 — Phylogenetic tree of Betaarterivirus americense based on GP5 sequences, generated using Nextclade v3.21.0. [file Image_2.pdf]

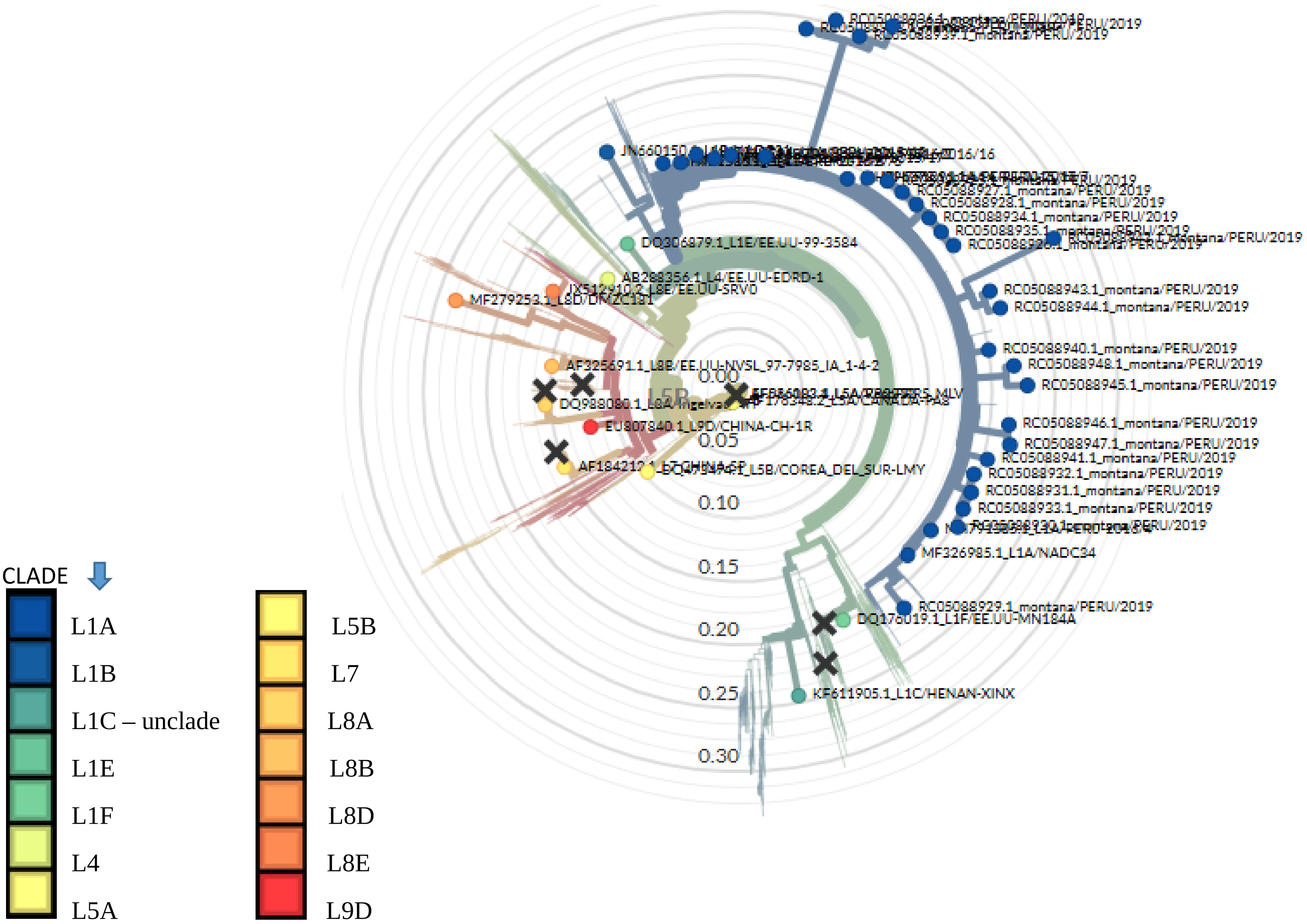

Supplementary Material 5. Phylogenetic tree of *Betaarterivirus americense* based on the glycoprotein 5 (GP5) sequence, generated using Nextclade v3.21.0 (https://clades.nextstrain.org/). Branches correspond to established lineages and are color-coded as follows: lineage 1—sublineage 1A (dark blue; MF326985.1_L1A/NADC34 and 24 study strains), sublineage 1B (light blue; JN660150.1_L1B/NADC31), sublineage 1C (sky blue; KF611905.1_L1C/HENAN-XINX), sublineage 1E (turquoise; DQ306879.1_L1E/US-99-3584), and sublineage 1F (forest green; DQ176019.1_L1F/US-MN184A); lineage 4 (lime green; AB288356.1_L4/US-EDRD-1); lineage 5—sublineage 5A (yellow; AF066183.4_L5A/RespPRRS_MLV and EF536003.1_L5A/VR2332); lineage 7 (yellow; AF184212.1_L7/CHINA-SP); lineage 8—sublineage 8A (light orange; DQ988080.1_L8A/Ingelvac_ATP); and lineage 9—sublineage 9D (EU807840.1_L9D/CHINA-CH-1R). All 24 strains analyzed in this study clustered within sublineage 1A (dark blue), with evident divergence among variants within the group.
